# Barriers to accessing paediatric critical care and surgery: A qualitative study

**DOI:** 10.1371/journal.pone.0355278

**Published:** 2026-08-04

**Authors:** Catherine El Zerbi, Emer Cullen, Elaine Bidmead, Helena-Ulrike Marambio, Rachel Agbeko, Julie Bloomfield, Judith Rankin

**Affiliations:** 1 Senior Research Fellow, Division of Health Research, Faculty of Health and Medicine, Lancaster University, Lancaster, United Kingdom; 2 Specialty Registrar in Public Health, Population Health Sciences Institute, Newcastle University, Newcastle upon Tyne, United Kingdom; 3 Senior Research Fellow, Institute of Health, University of Cumbria, Carlisle, Cumbria, United Kingdom; 4 National Institute for Health and Care Research (NIHR) Applied Research Collaboration (ARC) for the North East and North Cumbria, Newcastle upon Tyne, United Kingdom; 5 Assistant Professor, Institute for Legal Gender Studies, Johannes Kepler University, Linz, Austria; 6 Consultant Paediatric Intensivist, Great North Children’s Hospital, The Newcastle upon Tyne Hospitals NHS Foundation Trust, Newcastle upon Tyne, United Kingdom; 7 Associate Clinical Lecturer, Translational and Clinical Research Institute, Newcastle University, Newcastle upon Tyne, United Kingdom; 8 Network Manager, North East & North Cumbria Paediatric Critical Care & Surgery in Children Operational Delivery Network, South Tees NHS Foundation Trust, James Cook University Hospital, Middlesbrough, United Kingdom; 9 Professor of Maternal and Child Health, Population Health Sciences Institute, Newcastle University, Newcastle upon Tyne, United Kingdom; Universiti Sains Malaysia, MALAYSIA

## Abstract

**Background:**

Certain groups of children and young people (CYP) living in the North East and North Cumbria (NENC) present late to paediatric critical care (PCC) and surgery in children (SIC) services, resulting in poorer clinical outcomes. This suggests inequitable access; however, the factors influencing access to these services are presently unclear.

**Aim:**

To explore inequitable access to PCC and SIC for CYP and inform recommendations for service improvement.

**Design:**

Qualitative study using semi-structured interviews.

**Setting:**

Community settings in NENC, United Kingdom.

**Participants:**

CYP with experience of PCC/SIC, their caregivers, healthcare professionals, and charity staff.

**Analysis:**

Data were analysed using a combined deductive-inductive approach, informed by the UK Paediatric Critical Care Society Quality Standards.

**Results:**

Barriers to access were grouped into three categories: communication, system-level, and socio-economic factors. Communication and system-level barriers align with existing quality standards, whereas socio-economic influences are not explicitly addressed but play a significant role in shaping access for some families.

**Conclusion:**

Access to PCC and SIC is shaped by interacting communication, system, and socio-economic factors that may delay timely care. Greater recognition of socio-economic influences within service standards and policy may support more equitable access. Further research should focus on improving engagement with CYP and caregivers from underrepresented groups.

## Introduction

In England, Paediatric Critical Care (PCC) is designated as a *specialised* NHS service because it is a low-volume, high-cost service that is not available in every hospital. PCC is classified into three levels: *Level 1 (L1)*: Basic Critical Care; *Level 2 (L2)*: Intermediate Critical Care; and *Level 3 (L3)*: Advanced Critical Care [1]. Unlike most paediatric services, L3 PCC is only available in tertiary centres, which are predominantly located in urban areas and can be difficult to reach, particularly for those living in rural areas without easy access to transport [2]. Within the North East and North Cumbria (NENC), certain groups of children and young people (CYP) present late to PCC and surgery in children (SIC) services, including paediatric dental surgery, resulting in poorer clinical outcomes. This suggests inequities in access to these specialist healthcare services, however, the factors influencing access are presently unclear.

Poverty and low income are known factors influencing CYP’s health [3]. CYP living in areas of high economic deprivation have increased rates of attendance at Emergency Departments (ED) and higher rates of unplanned hospital admissions than those from less economically deprived areas [4,5]. Socio-economic deprivation is also associated with an increased risk of admission to PCC [6,7], poorer clinical outcomes [8] and emergency readmissions [4]. The NENC region has high levels of economic deprivation [9]. Eight out of the ten local authorities with the highest rates of child poverty are in the NENC, with 35% of children in the NENC living in poverty, ranging from 24.5% (North Tyneside) to 38.7% (Middlesbrough) [10]. If existing poverty levels remain, future projections of healthcare activity suggest that by 2040, infant hospital admissions are set to increase by 58%, and ED attendance by 144% [11]. Waiting lists for children’s health services are also increasing at double the rate of adult waiting lists. Children are more likely than adults to have to wait for over a year to be seen by medical, surgical and community paediatric teams, suggesting inequities between adult and children’s health services [12].

Reducing inequities related to accessing healthcare services has been an aim for the NHS for decades, including addressing barriers to timely access to healthcare. While the NHS provides universal healthcare free at the point of access, many potential barriers to timely access have been identified [13] including proximity to services and transport costs [4], working in insecure/inflexible employment, ethnic background [14], previous bad experiences, lack of access to primary care services, and in some reports, less timely recognition of illness [5]. The NHS identify that health services must adapt to meet the changing needs of patients, and that high quality care, as close to home as possible, should be provided for all CYP [15]. To achieve this, it is important to understand the needs of the population and barriers to accessing healthcare services [16], which is a key element to achieving equity within a health system [17]. While much is known about barriers to health services [18,19], little evidence exists on barriers to accessing PCC and SIC. Insights into the barriers could have relevance to other socio-economically deprived regions of the UK. Identifying and understanding these barriers will enable service improvements to these vital services, which in turn could lead to improvements in health outcomes for CYP and families.

### Aim

To explore inequitable access to PCC and SIC for CYP in NENC and inform service improvement.

## Materials and methods

### Design

We conducted a qualitative study using semi-structured interviews, analysed using a combined inductive-deductive approach informed by The UK Paediatric Critical Care Society Quality Standards for the Care of Critically Ill or Injured Children [1]. A qualitative study design was selected to enable us to explore different perspectives on accessing PCC and SIC, and specifically how access might be socially shaped, context-dependent, influenced by systems, relationships, and lived experience. Reporting of our study was written in accordance with the ‘Consolidated criteria for reporting qualitative research’ (‘COREQ’) checklist [20] (S1 Table 1).

### Setting

Our study was based in the NENC region where PCC and SIC are delivered by eight NHS Foundation Trusts. Three levels of PCC are provided in the region; six Trusts provide inpatient paediatric care, one Trust has a L2 unit, and one Trust provides L2 and L3 critical care. Several factors shape the rate at which CYP are admitted to a L3 Paediatric Intensive Care Unit (PICU). These include: local levels of socio-economic deprivation, ethnicity, rate of significant comorbidities, and the quality of primary, secondary and tertiary care available. Further influences include system and process factors such as differences between L3 providers on the level of threshold for PCC admission [21].

In terms of demographics, at the time of the study, the ethnic make-up of the NE and NC was predominantly white British (90.6% and 95.2% respectively) compared to England and Wales as a whole (74.4%) [22]. While we advertised participation opportunities widely to diverse populations through VCSE and primary care networks, given our budgetary and capacity constraints, we were unable to produce bespoke recruitment methods, including multilingual materials, as well as multilingual staff, to engage with underrepresented communities. It is unclear therefore whether our findings will have relevance to regions with greater ethnic diversity.

### Ethics approval

This study was ethically reviewed and approved by the North East – Tyne & Wear South NHS Research Ethics Committee (study reference 313965), part of the Health Research Authority in England. Electronic or written informed consent was obtained from all participants prior to data collection. For participants under 18 years of age, parental consent was obtained, together with assent from the young person. Participants consented to the use of anonymised interview excerpts in academic dissemination, including peer-reviewed publications.

### Participants

Demographics, specifically age, ethnicity and first three characters of postcode, were collected for descriptive purposes and to monitor sampling strategies. Participants were:

1. CYP aged 12–16 years admitted to NENC PCC and SIC services for all conditions within the past six months from date of study entry;
2. Primary caregivers of CYP aged 0–18, whose child had been admitted to NENC PCC and/or SIC services for all conditions within the past six months from date of study entry;
3. HCPs working in NENC PCC or SIC, and VCSE staff working alongside NENC PCC or SIC services.

See S2 Table for participant inclusion and exclusion criteria. We restricted the ages of CYP participants to 12–16 years as PICUs typically care for children from birth up to their 16^th^ birthday [23]. Some PICUs make exceptions for young people up to 18 years old, with those aged over 16 years having an option to choose whether to be seen in the paediatric or adult area. The lower limit was 12 years, an age at which children are often considered capable of providing assent to participate in research, in addition to legal guardian consent.

### Patient and public involvement

Participant information sheets and interview topic guides for CYP were reviewed for accessibility and face validity by a young advisor from the NIHR Applied Research Collaboration NENC Young People’s Advisory Network to ensure the questions were clear and relevant to CYP's experiences. For content validity, senior clinicians in PCC and SIC advised on the staff topic guides, which were then piloted and refined by the research team. After analysis, preliminary themes were discussed with HCPs, caregivers, and VCSE staff for relevance.

### Recruitment

Community-based recruitment strategies were employed (S3 Table). CYP, caregivers, HCPs, and VCSE staff were invited to participate using convenience and snowball sampling. Direct recruitment from PCC and SIC was avoided for ethical reasons, as we did not want to disturb CYP and caregivers during hospital visits for serious health conditions. Instead, hospital partners displayed study adverts in paediatric critical care and surgery units to raise awareness among staff and potential participants. VCSE collaborators also shared electronic adverts with their networks, inviting interested individuals to contact the research team. Awareness was further spread through NHS trusts and regional health networks via newsletters and social media.

Potential participants self-referred on an opt-in basis using the contact details provided. Upon expressing interest, they were emailed an information sheet to review carefully, followed by a request for electronic or written informed consent from all participants, including legal guardians for those under 16. Participants were given verbal support during interviews if needed, and verbal consent was sought to continue. None of the participants withdrew from the study. All participants received a debrief leaflet with mental health support resources and a £20 shopping voucher as thanks for their contribution.

### Methods

Data collection occurred between 22^nd^ July 2022 and 31^st^ August 2023. Data were collected through a choice of one-to-one or paired semi-structured interviews for CYP, and interview or focus group for caregivers. PCC and SIC staff, as well as VCSE staff, were invited to participate via semi-structured interviews. Interviews occurred once and were not repeated due to limited funding and capacity. All participants were given the option to be interviewed either face-to face, via video (using Microsoft Teams), or telephone. Interviews lasted on average 45 mins, and were conducted by one of two female researchers (CE, EC), with no one else present. Participants were not known to the researchers conducting the interview. At the time of the study, the lead interviewer (CE) was a postdoctoral fellow with over a decade of experience in qualitative applied health research, and the second interviewer (EC) was a medically qualified registrar completing advanced training in public health.

The UK Paediatric Critical Care Society Quality Standards for the Care of Critically Ill or Injured Children [1] were used as a theoretical framework to guide and provide consistency across interviews. We specifically drew on the quality standards for *Level 3 Paediatric Critical Care Units on information and support for children and their families*, which was incorporated into our topic guides (S4 Table). The standards outline what PCC services should be providing and the relevant standards for doing so. Where a service does not meet or deliver these standards, it is recommended that a risk analysis is completed. As such, the standards can be used by any service as a framework for local improvements. We judged these standards to be the most suitable guiding framework to drive quality improvement in PCC and SIC services, including Specialist Paediatric Transport Services.

### Analysis

Interviews were audio-recorded and transcribed verbatim, with qualitative data subsequently managed using NVivo Version 13 software [24]. Transcripts were not returned to participants for participant validation (member checking), nor were participants invited to provide feedback on the findings. We intentionally refrained from defining the concept of ‘access’ a priori, allowing meanings to emerge during analysis based on participants’ first-hand insights and experiences with health services. Our epistemological stance was informed by Dixon-Woods and colleagues’ concept of ‘candidacy’ [18], which highlights how individuals’ eligibility for medical attention and intervention is negotiated between themselves and health services. This perspective enabled us to view access to PCC and SIC services as a dynamic, situated, and layered process. Consequently, we recognise that not all individuals experience barriers in the same way. Formal comparative analysis between groups was not undertaken at this early stage. Instead, our combined deductive-inductive framework analysis (see below) prioritised identifying cross-cutting themes relevant across participant groups.

Data collection and analysis were conducted iteratively using a combined deductive-inductive approach which drew on best-fit framework analysis [25–27] allowing us to explore both anticipated and emergent themes. Best-fit framework analysis is a qualitative approach that combines deductive and inductive logic. It extends traditional framework analysis [28–29] by enabling the use of an a priori conceptual framework to guide data interpretation while retaining flexibility to incorporate emergent findings. Originally developed for qualitative evidence synthesis, this approach is increasingly applied in primary qualitative research, particularly in studies of patient and caregiver experiences of barriers to care [30–31].

We used the UK Paediatric Critical Care Society Quality Standards for the Care of Critically Ill or Injured Children PCCS [1] as a framework to structure initial coding across different participant groups, while also supporting the generation of policy-relevant insights grounded in an established structure. Analysis proceeded in two stages (S5 Figure): first, deductive coding was undertaken using domains derived from our framework; second, inductive coding was conducted to identify novel or unanticipated barriers emerging from the data, with iterative refinement of the analytic framework. CE coded all transcripts line-by-line while making detailed and reflexive annotations to track inductive thematic development. To facilitate alignment between the data, researcher coding and the quality standards framework, EC and HM each coded three transcripts which were discussed with CE for comparison, consensus and refinement of initial codes.

Analysis was conducted concurrently with recruitment. We continued with recruitment until sufficient depth and diversity of perspectives were achieved to address the research question (i.e., thematic sufficiency). A coding framework (S6 Table) was created by CE to record major and minor themes for each participant group, which were then discussed in analytical meetings. Based on these discussions, codes were then indexed, charted, mapped, interpreted and developed into main themes by CE, then named and defined together with illustrative extracts. CE and EB then reviewed illustrative extracts in conjunction with CE’s coding annotations to assess whether the proposed themes deductively ‘fit’ existing domains of the quality standards. For codes that did not fit the framework, or those we considered to have emerged ‘inductively’, ongoing discussions were held to conceptually expand the framework where required. Illustrative extracts were then considered and selected for each theme (S7 Table).

## Results

Seventeen participants were included (Table 1). Three paired interviews were conducted (two with a young person and caregiver [n = 4]; one with two caregivers who were also VCSE workers [n = 2]), with the remaining participants interviewed individually (n = 11). Three major themes were identified: communication barriers; PCC and SIC system barriers; and the need to expand the PCC quality standards framework to incorporate socio-economic barriers. Themes are illustrated with verbatim quotes, alongside participant status (Young Person [YP], Caregiver, HCP, VCSE) and setting (PCC, SIC, paediatric dental surgery [PDS], emergency department [ED]).

**Table 1:** Summary of participant characteristics.

Characteristic	N = 17
**Setting:**	
SIC	4
PCC	12
Dental surgery	1
**1. Child or young person:**	2
*12 years*	2
**2. Caregivers**	6
*- Mother*	*4*
*- Father*	*2*
**3. Healthcare staff**	6
- Consultant	3
- Nurse	3
**4. VCSE staff**	3
Ethnicity*:	
- White British	15
- Ethnic Minorities*	2
Area**:	
*- Cumbria*	2
*- Durham*	3
*- Hartlepool*	4
*- Newcastle*	7
*- Sunderland*	1

*Ethnicity data removed as potentially identifiable

### 1. Communication barriers.

We defined communication barriers as those related to approaching, accessing, understanding and expressing key information on healthcare, and challenges in reporting health conditions [32]. This included communication difficulties between a child, YP or caregiver in their interactions with a HCP, disagreements in clinical decision-making processes between clinicians, and/or between hospital departments/units, between hospitals (e.g., district general hospital (DGH) and tertiary centre), and between sectors (e.g., health and social care). Communication barriers were reported by young participants and their caregivers, described by one young participant as a struggle to speak the ‘*right*’ words to articulate their level of pain to HCPs. While positive instances of communication were described with individual members of staff, a sense of exclusion from medical decisions, feeling dismissed when asking questions, and a lack of reciprocity were brought to the fore. The importance of caregivers having the confidence to “*advocate*” for their child was highlighted, with those unable to speak up perceived as “*just being completely trampled*”, “*completely overwhelmed*”, “*absolutely ignored*”, or left feeling that “*your opinion doesn’t matter*”, as one father reported. Caregivers perceived “*dismissiveness*” from healthcare staff – which they attributed to working in a busy clinical environment. This compounded their lack of understanding and control, and caregivers felt there was an expectation of them to “*just sign the consent form*”.

### 2. PCC and SIC system barriers.

These barriers related to system design and infrastructure, which we further subdivided into *structural resource deficiencies* and *operational process failures*, with overlap across both. Structural resource deficiencies included underfunding and understaffing, lack of specialist paediatric clinical staff, location and geography of DGH and distance to tertiary centre. Operational process failures included delays in transfer process between hospitals, lack of follow-up by hospital, difficult care pathways and poor care co-ordination, physical obstructions to accessing hospital, especially for those who are ill and/or with disabilities, lack of provision of age-appropriate healthcare information for patients and families, prolonged waiting lists, and insufficient space and beds on units. Distance, specifically the lack of access to higher-level care in one geographically remote DGH, was perceived by HCPs to result in worse clinical outcomes for CYP due to delays in receiving specialist paediatric care. Staffing was also a concern in hospitals. For example, one participant working in a PICU mentioned that understaffing meant they had to take on additional roles to cover gaps. In terms of barriers to surgery, there were concerns about delays due to a lack of paediatric surgeons in one DGH. Inappropriate staffing and understaffing were seen as “*impairing*” the quality of care for CYP living in geographically remote and less populated areas. Lack of access to a specialist paediatric intensivist, anaesthetic and surgical staff in geographically remote and less populated areas meant that when a child or YP was seriously ill, they needed to be transferred to a PICU in a tertiary centre. While beds were reportedly available in PICU, often the challenge was arranging transfers by specialist teams for critically ill patients to tertiary centres from geographically isolated DGHs. Some HCPs highlighted how tertiary centres appeared to be “*reluctant*” to collect CYP patients, despite the urgency. These delays were reported to cause “*a lot of distress*” for caregivers and HCPs, and as such, it is important for tertiary centres to recognise the pressures faced by colleagues in DGHs, particularly in the absence of specialist paediatric staff and resource. Waiting times, at the time of interviews, were particularly problematic and were reported to have resulted in late diagnoses, and health conditions deteriorating, which negatively affected CYP’s quality of life.

Barriers specific to paediatric dental surgery included a perception that dental waiting lists were not seen as a priority compared to other medical specialities and subsequently were cancelled to accommodate non-dental surgeries. Delays in access were also reported for paediatric dental patients with medically complex conditions who required access to specialists for treatment planning, as well as patients with learning disabilities and neurodiversity who required specially trained staff. Caregivers and VCSE staff perceived a lack of disability awareness in the NHS as contributing to inequities in access and existing health inequalities for CYP with disabilities. A lack of joined up systems between PCC and social services was referred to as a “*blockage*” to understanding socio-economic barriers to access. A lack of access to social care data prevented HCPs from proactively addressing how potential socio-economic disadvantages may adversely impact access to healthcare and health outcomes.

### 3. Socio-economic barriers.

These barriers related to the socio-economic and financial circumstances of patients and caregivers, including costs associated with attending hospital, income status, employment conditions, childcare provision, and a caregivers’ ability to recognise the severity or signs of deterioration of their child’s health condition. Such costs were especially challenging for caregivers living on low incomes. Some caregivers missed work due to sleeplessness, exhaustion and the stress associated with their child's condition; some were concerned about losing their job, and others described needing to stop working altogether, with careers halted due to full-time caring responsibilities. HCPs also perceived some CYP to be disadvantaged by a caregivers’ inability to recognise the severity or signs of deterioration of their child’s health condition, resulting in delayed access.

## Discussion

Our findings identified communication, system, and socio-economic barriers as significant obstacles to accessing PCC and SIC in the NENC. While communication and system-level barriers are addressed in the quality care standards established by the Paediatric Critical Care Society [1], socio-economic barriers are not currently included. Notably, our study revealed the substantial role socio-economic factors play in shaping access to PCC and SIC for certain families. These findings align with Dixon-Woods and colleagues’ concept of ‘candidacy’ [18], which posits that accessing PCC and SIC is a dynamic process negotiated between individuals, families, and health services. This perspective highlights that ‘accomplishing’ access to healthcare can be complex for many families, requiring effective communication with HCPs, a competent and responsive local health system, and the practical resources necessary for CYP and their families to physically reach the hospital.

In relation to ***communication barriers***, our younger participants and their caregivers talked about an unequal dynamic in their encounters with *some* HCPs. They also expressed difficulty in finding the words to articulate their pain in a way that was understood. Caregivers described needing a certain set of competencies to be able to understand (and consent to) complex medical procedures, and a confident ‘voice’ to be able to advocate for their child and/or question medical authority when unclear or in doubt. As set out in the NHS constitution and professional standards [33–35], it is important that HCPs involve CYP and caregivers in decisions about their healthcare in ways that are appropriate to their maturity and understanding. While a recent review [36] indicated a lack of high-quality research on the best methods for healthcare communication with CYP, it did highlight the importance of an early, clear and accessible approach to medical communication, one that is non-judgmental, welcoming, open-minded, supportive, encouraging and adaptive to individual needs. This aligns with a large body of literature on childhood calling for the adoption of a rights-based and inclusive approach to involving CYP in healthcare decision-making [37].

Our findings indicated that ***system barriers*** disproportionately affected CYP living in rural areas. This matters because HCPs expressed concern that distance barriers put patients at greater risk for poorer outcomes compared to those living closer to tertiary centres. For instance, in NC, the distance to tertiary care facilities was significant, creating substantial challenges for low-income families, particularly those in remote locations, with inflexible employment, poor physical or mental health, and limited access to transportation. Previous research has shown that CYP from low-income backgrounds referred to tertiary care are less likely to attend scheduled appointments [38]. In the UK, PCC transport teams aim to reach critically ill children within three hours of a decision for intensive care. However, intubation is required for PICU admission, and transport teams are not obligated to accept non-intubated patients. This design relates to different levels of PCC: patients needing Level 3 (L3) critical care are transported to PICUs, while Level 1 and 2 do not have equivalent arrangements. Transfer and admission challenges for CYP in geographically isolated DGHs were noted, with delays stemming from disagreements among HCPs about clinical thresholds for transfer. While it remains unclear how these factors impact patient health outcomes, one study found a slight increase in PICU length of stay for children awaiting transport [39]. Additionally, our research demonstrated that system-level barriers, such as difficulties in obtaining appointments or referrals, hindered caregivers’ ability to access services in a timely manner, irrespective of socio-economic status. Dixon-Woods and colleagues [18] use the term ‘permeability’ to describe the ease with which individuals access healthcare services. A ‘porous’ service, like EDs, requires fewer resources to access, whereas less porous services, such as PCC and SIC, necessitate referrals and appointments. Delays in obtaining referrals and surgical appointments were identified in this study, highlighting the less permeable nature of PCC and SIC as specialist healthcare services.

A key finding was the impact of socio-economic conditions on access to PCC and SIC, consistent with previous research [2,4,5]. ***Socio-economic barriers*** were characterised by a lack of practical resources, such as transportation to the hospital, difficulty in taking time off work, and the financial costs associated with missed work, mirroring previously identified obstacles to healthcare access [40]. Additionally, HCPs noted that caregivers’ ability to recognise and understand warning signs of a child's deterioration can be limited, potentially delaying timely access to hospital services. This issue aligns with the concept of ‘health literacy,’ [41] defined as the skills and social resources individuals need to access, understand, appraise, and use health information and services. Our findings support earlier studies indicating that individuals in socio-economically disadvantaged circumstances are less likely to seek healthcare early, more likely to underutilise preventive services [42], and tend to downplay warning signs until reaching a crisis [18], thereby delaying access to care. The overall experience of PCC and SIC, especially the unaffordability of the associated costs for some, added to the stress, fear, worry, fatigue and exhaustion, poor nutrition and trauma experienced by caregivers, with lasting effects on mental health, employment and careers; consistent with previous reports [43–44].

### Limitations

Several methodological challenges were encountered in this study, all of which limit the extent to which any inferences can be drawn. Firstly, difficulties in recruiting all participant groups resulted in a high likelihood that important views on barriers to access are missing. Only two YP were recruited, meaning that barriers from the perspectives of CYP remain poorly understood, a factor to consider when interpreting the findings. Given the small and uneven sample sizes within our subgroups, we considered that subgroup comparisons would not be methodologically robust and may risk over-interpretation of context-specific perspectives. This means transferability of findings to wider populations is limited and differences between groups may be overstated or understated. Additionally, the external validity and generalisability of our results to other populations and regions are limited due to our use of convenience and snowball sampling. While the insights generated may be relevant to other socio-economically underserved regions of the UK, our focus on a small subset of experiences limits the applicability of identified barriers to other contexts. Pragmatic constraints, including limited time and funding, impacted our ability to implement more robust and engaging recruitment strategies.

Secondly, although multiple recruitment strategies were employed resulting in a rich dataset with conceptual depth, the low sample size prevented us from reaching ‘data saturation’, particularly for CYP. Again, this limitation suggests that important issues or insights may have been missed. Third, it is possible that the time lag between their hospital experiences and study participation may have impacted participants’ ability to recall certain details, although this seems unlikely given the richness and depth of participants’ descriptions. Fourth, given the exploratory nature of the study, CYP and caregivers facing specific medical conditions and/or financial hardships were not specifically targeted, despite financial constraints often being raised by participants. Fifth, although the identification of socio-economic factors was a key finding, our analysis remained superficial (e.g., focusing on transport and parking costs). Finally, interviews were conducted with English-speaking participants, leaving the experiences of CYP from families where English is not spoken unexplored. Views on barriers to access from ethnically diverse people and communities are underrepresented in this study, further limiting the generalisability of our findings to the broader population.

### Key learning for future research

Our study identified several key considerations for future research:

1. **Co-design with stakeholders**: CYP, their caregivers, and VCSE staff should be invited to co-design the study as equal partners. Their active involvement from the pre-application phase through all stages of the research process will enhance feasibility, acceptability, efficiency, and relevance.
2. **Diverse participation**: Effective community engagement and recruitment strategies are essential. Future research with larger and more balanced samples could usefully explore differences between participant groups to further refine understanding of how barriers may differentially affect populations. There is an urgent need to engage CYP and their caregivers with experiences of PCC and SIC, particularly those with complex health needs, from low-income backgrounds, geographically remote areas, and ethnic minority groups. Future studies should explore how socio-economic status intersects with geographical isolation, disability, and race, leading to compounded inequalities. Researchers should collaborate with local representatives from underrepresented communities to better understand their needs and barriers to health research participation. Study designs should include multilingual materials and staff, widely advertise participation opportunities, and allocate budget and time for community engagement throughout the research process.
3. **Enhancing study awareness**: To improve recruitment, public awareness of study participation should be increased among CYP by creating accessible videos for social media and distributing study adverts in patient discharge letters and local media outlets. VCSE participants suggested presenting the study and distributing adverts at school and community events, particularly those for special educational needs and disabilities.
4. **Inclusion of experienced hospital staff**: Given the complexity and ethical sensitivity of PCC and SIC research, study teams should include paediatric research nurses with relevant expertise.
5. **Innovative research methods**: In light of communication challenges, it is vital to co-create developmentally appropriate, interactive tools, such as puppets, books, drawings, apps, and videos, to enhance CYP’s understanding of the research and enable them to express their views. VCSE participants also recommended involving an experienced play therapist to assist with data collection.

### Policy and practice recommendations

Twenty-two evidence-informed recommendations have been established to improve barriers in PCC systems [20]. Our findings support these recommendations, particularly aligning paediatric care pathways more closely; increasing the number of L2 beds to reduce strain on L3 beds; ensuring capacity matches demand; and ensuring adequate clinical staff are available to meet demand, possessing relevant critical care expertise. In addition, we recommend the following:

1. **Provision of age-appropriate information:** Local health systems should provide PCC and SIC services with age-appropriate, engaging, and accessible information for CYP and their caregivers prior to hospital visits. This information should address expectations, roles, and the risks and benefits of care, and be co-designed with input from CYP, caregivers, and VCSE staff.
2. **Awareness of early warning signs:** Local health systems should enhance caregivers’ knowledge of early warning signs of clinical deterioration at home by raising awareness of existing tools, such as the NHS Healthier Together website and app [45], which provide guidance and reassurance.
3. **Financial support signposting:** HCPs or hospital social workers should proactively signpost caregivers to financial support for costs associated with PCC and SIC, including transport, parking, sustenance, accommodation, and necessary equipment. Establishing data-sharing agreements between hospitals and social services could help identify broader determinants affecting CYP's access to healthcare.
4. **Development of support policies:** Local health systems must address the urgent lack of policies to support caregivers when their child attends PICU and/or SIC, as this is crucial for safeguarding caregivers’ health.

## Conclusion

Our findings underscore the challenges families face in achieving access to specialised healthcare, highlighting that this process can be difficult and complex. Successful access requires effective communication with HCPs, a local health system that is both competent and responsive, and practical resources to enable families to physically reach the hospital. We acknowledge limitations related to sampling and recruitment in our study. Consequently, before undertaking a future study, it is essential to develop effective recruitment strategies aimed at engaging CYP and their caregivers with experiences in PCC and SIC, particularly those with complex health needs, and from low-income backgrounds, geographically remote areas, and ethnic minority groups. Overall, this study provides valuable insights for informing future research directions and identifies critical areas for PCC and SIC providers to address to promote equitable access to care.

## Supporting information

S1 Table COREQ. (DOCX)

S2 Table Inclusion and exclusion criteria. (DOCX)

S3 Table Recruitment strategies. (DOCX)

S4 Table Topic guides for interviews. (DOCX)

S5 Figure Deductive-inductive analytical steps. (DOCX)

S6 Table Deductive-inductive coding framework. (DOCX)

S7 Table Illustrative extracts. (DOCX)
