# Supplementary material for: Barriers to accessing paediatric critical care and surgery: A qualitative study
Source: PLoS One. 2026 Aug 4;21(8):e0355278. doi: 10.1371/journal.pone.0355278 (PMC13436771; doi:10.1371/journal.pone.0355278)
Supplement: Supplementary file 2 [file pone.0355278.s002.docx]

**S2 Table 1.** Inclusion and exclusion criteria.

| **Participant group** | **Definition** | **Inclusion criteria** | **Exclusion criteria** |
| --- | --- | --- | --- |
| **Group 1. Children & young people** | Refers to a child or young person aged 12 to 16 years. Critically ill and critically injured children and young people may have been referred to paediatric critical care whether medically, surgically or trauma-related. Critically ill and critically injured children and young people may present in Emergency Departments, Children’s Assessment Services or become critically ill whilst in in-patient children’s services. Those needing an enhanced level of observation, monitoring or intervention will need to be taken to a Paediatric Intensive Care Unit. | - Gender: Male, female and non-binary participants  - Lower age limit: 12 years;  - Upper age limit: 16 years  - Have attended paediatric critical care and surgery in children departments across the NENC within the past six months for all conditions | - CYP who are current inpatients in paediatric critical care or surgery departments  - Children under 12 years old  - Severe psychological distress or cognitive impairment likely to preclude participation  - Child or young person, or legal guardian, unable to provide informed consent |
| **Group 2. Primary caregivers** | Includes parents, siblings, grandparents, extended family members or others with carer responsibility. | - Gender: Male, female and non-binary participants  - Lower age limit: 18; Upper age limit: no upper limit  - Primary caregivers of children and young people aged 0-18 years who have attended paediatric critical care and surgery in children departments across the NENC within the past six months | - Primary caregivers of children and young people who are current inpatients in paediatric critical care or surgery in children departments  - Severe psychological distress or cognitive impairment likely to preclude participation  - Caregiver unable to provide informed consent |
| **Group 3. Healthcare workers** | Refers to a registered healthcare professional. | - Gender: Male, female and non-binary participants  - Lower age limit: 18; Upper age limit: no upper limit  - Healthcare staff currently working in paediatric critical care and surgery in children departments across the NENC  - Ability to read and write English | - Staff who have not worked in paediatric critical care or surgery in children departments in the last year |
| **Group 4. VCSE workers** | Refers to staff working in communities and the charity, voluntary and social enterprise sector that were supporting paediatric critical care and surgery services. | - Gender: Male, female and non-binary participants  - Lower age limit: 18; Upper age limit: no upper limit  - Staff working in the VCSE sector and the community who supported paediatric critical care and surgery departments across the NENC  - Had direct experience supporting families in the last six months  - Ability to read and write English | - VCSE staff who have not had direct experience supporting families in the last year |
