# Supplementary material for: Barriers to accessing paediatric critical care and surgery: A qualitative study
Source: PLoS One. 2026 Aug 4;21(8):e0355278. doi: 10.1371/journal.pone.0355278 (PMC13436771; doi:10.1371/journal.pone.0355278)
Supplement: Supplementary file 3 [file pone.0355278.s003.docx]

**S3 Table 1.** Recruitment strategies.

| **Group** | **Recruitment strategies** |
| --- | --- |
| **i. Children & Young People** | 1. Young people (and legal guardians where under 16 years) attending NHS clinics (e.g. general paediatrics, speciality paediatrics) following critical care or surgery will be provided with paper copies of a study advert and/or participant information sheet by hospital staff (e.g. research nurses) which included telephone, email and address contact details for the research team, who they were asked to contact if they were interested in taking part.  2. Hospital partners displayed a study poster on their public noticeboards with contact details for the research team.  3. Hospital partners facilitated face-to-face consultations - if desired - between interested individuals and a member of the research team following study information sharing.    4. VCSE organisations affiliated with paediatric critical care and surgery in children across region were be approached by the research team to share study adverts with young people.  5. Special educational needs and disabilities (SEND) schools across region were approached by the research team to share study adverts with children and caregivers. |
| **ii. Caregivers** | 1. Caregivers of young people attending NHS clinics following critical care or surgery were provided with paper copies of a participant information sheet by hospital staff which included the telephone, email and address contact details for the research team, who they were asked to contact if they were interested in taking part.  2. Hospital partners displayed a study advert on their public noticeboards with contact details for the research team and distributed a study advert in patient discharge and follow-up letters.  3. VCSE organisations affiliated with paediatric critical care and surgery in children across region were approached by the research team to share study adverts with caregivers.  4. Facebook groups for carers were approached by the researchers to share study adverts with their members.  5. GP surgeries and pharmacies across the North East and North Cumbria were approached to display study adverts on their public noticeboards, and digitally via newsletters and bulletins. |
| **iii. Hospital staff** | 1. Hospital partners displayed a study advert on staff noticeboards in paediatric critical care and surgery units to make paediatric, surgical, anaesthetic and critical care staff aware of the study.  2. Staff were emailed a study advert and participant information sheet by hospital partners and were asked to contact the research team if they were interested in taking part. |
| **iv. VCSE staff** | 1. VCSE organisations working alongside paediatric critical care & surgery services across region were identified via desk-based searches and hospital partners. A manager was identified by the research team who was then asked to distribute paper and electronic versions of a study advert and participant information sheet to staff. |
| **v. All** | 1. Following interviews, group activities or focus groups, participants were asked if they were aware of any potential informants who they could share study information with (snowball sampling). |
