# Supplementary material for: Barriers to accessing paediatric critical care and surgery: A qualitative study
Source: PLoS One. 2026 Aug 4;21(8):e0355278. doi: 10.1371/journal.pone.0355278 (PMC13436771; doi:10.1371/journal.pone.0355278)
Supplement: Supplementary file 4 [file pone.0355278.s004.docx]

**S4 Table 1.** Topic guides for interviews.

| **Participant group** | **Summary topics for interviews** | **Alignment with UK PCC Quality Standard** |
| --- | --- | --- |
| ***i. Children, young people & caregivers*** | i. Part of region accessed hospital. ii. Description of health condition. iii. Ease of access to healthcare in local community. iv. Clinical journey/referral pathways to hospital admission in the past six months. v. Transport to hospital. vi. Communication with hospital. vii. Clarity of information provided by hospital about admission. viii. Understanding of hospital process. ix. Interactions with hospital staff. x. Feelings, experiences and perceptions about hospital stay. xi. Facilities & support for caregivers provided at hospital. xii. Awareness of any inequities in access from witnessing or first-hand experience. xiii. Impact of barriers on the child/young person/caregiver, and community in general. xiv. Improvements for hospitals/what is needed. xv. Anything else on barriers or challenges. | **L3-101 Child-Friendly Environment**  Children should be cared for in a defined safe and secure child-friendly environment, with age-appropriate stimulation and distraction activities.  **L3-102 Parental Access and Involvement**  Parents should:  a. Always have access to their child except when this is not in the interest of the child and family or of the privacy and confidentiality of other children and their families  b. Be informed of the child’s condition, care plan and emergency transfer (if necessary) and this information should be updated regularly  c. Have information, encouragement and support to enable them fully to participate in decisions about, and in the care of, their child.  **L3-103 Information for Children & Young People**  Children and young people should be offered age-appropriate information, encouragement and support to enable them to share in decisions about their care. Written information about common conditions should be available.  **L3-104 Information for Families**  Information for families should be available covering, at least:  a. The child’s condition  b. How decisions are made and how parents should be involved in decisions relating to their child’s care  c. Participation in the delivery of care and presence during interventions  d. Support available including access to psychological and financial support  e. How to get a drink and food  f. Layout of the unit or ward, visiting arrangements including arrangements for children to visit, car parking advice, ward routines and location of facilities within the hospital that families may wish to use  g. Relevant support groups and voluntary organisations.  **L3-105 Facilities and Support for Families**  Facilities should be available for families, including:  a. Somewhere to sit away from the ward  b. Quiet room for sensitive discussions with healthcare professionals  c. Kitchen, toilet and washing area  d. Changing area for other young children  e. Midwifery and breast-feeding support  f. Breast feeding facilities  g. Chair for parents to sit next to the child  h. Access to psychological support.  **L3-196 Discharge Information**  On discharge home, children and families should be offered written information about:  a. Care after discharge  b. Early warning signs of problems and what to do if these occur  c. Who to contact for advice including contact details.  **L3-197 Additional Support for Families**  Families should have access to the following support and information about these services which should be available:  a. Interfaith and spiritual support  b. Social workers  c. Interpreters  d. Bereavement support  e. Patient Advice and Advocacy Services.  **L3-199 Involving Children and Families**  The service should have:  a. Mechanisms for receiving feedback from children and families about the treatment and care they receive  b. Mechanisms for involving children and families in decisions about the organisation of the service  c. Examples of changes made as a result of feedback and involvement of children and families. |
| ***ii. Healthcare staff*** | i. Job role and years of experience. ii. Background and context of ward/unit. iii. Needs of specific groups accessing services. iv. Referral pathways to ward/unit. v. Lists and waiting times. vi. Consideration for caregivers when offering appointments. vii. Views on information provision for CYP and caregivers. viii. Approach to informed to requesting informed consent. ix. Approach to rights and reasonable adjustments for CYP and caregivers. x. Facilities & support for caregivers on ward/unit. xi. Involving CYP and caregivers in clinical decisions. xii. Debrief and discharge process. xiii. Mechanisms for feedback from CYP and caregivers. xiv. Awareness, views, experiences and perceptions of barriers to access to PICU and SIC services for CYP and caregivers (e.g. indicators of difficulty, signposting to help, interventions). xv. Impact of barriers on the child/young person/caregiver, and community in general. i. Improvements for hospitals/what is needed. ii. Anything else on barriers or challenges. | **L3-101 Child-Friendly Environment**  **L3-102 Parental Access and Involvement**  **L3-103 Information for Children & Young People**  **L3-104 Information for Families**  **L3-105 Facilities and Support for Families**  **L3-196 Discharge Information**  **L3-197 Additional Support for Families**  **L3-199 Involving Children and Families** |
| ***iii. VCSE staff*** | i. Job role and experience. ii. Connection to PICU and SIC services. i. Background and context of organisation/group and needs addressed or individuals/communities served. ii. Referrals process to organisation/group. iii. Awareness, perception, views and experiences to barriers to PIC and SIC if any, encountered by communities. iv. Infrastructure, if any, in community to support access to PIC and SIC. v. Impact of barriers on the child/young person/caregiver, and community in general? i. Improvements for hospitals/what is needed. ii. Anything else on barriers or challenges. | **L3-103 Information for Children & Young People**  **L3-104 Information for Families**  **L3-105 Facilities and Support for Families**  **L3-196 Discharge Information**  **L3-197 Additional Support for Families** |
