# Supplementary material for: Barriers to accessing paediatric critical care and surgery: A qualitative study
Source: PLoS One. 2026 Aug 4;21(8):e0355278. doi: 10.1371/journal.pone.0355278 (PMC13436771; doi:10.1371/journal.pone.0355278)
Supplement: Supplementary file 5 [file pone.0355278.s005.docx]

**S5 Figure 1.** Deductive-inductive analytical steps.

Step 1.

Select an existing theoretical framework or model appropriate to the setting and population

Deductive

Step 2.

Familiarise yourself with the data, create a matrix using the domains of the framework, and code using the framework in the initial instance

Inductive

Step 3.

Add new codes - as you go, create new codes when data don’t fit your existing framework for new insights

This is where unexpected insights emerge

Step 4.

Refine and merge codes to create themes - review all codes, combine similar ones, split overly broad ones, and organise into meaningful groups

Step 5.

Create new themes if extracted data cannot be coded into chosen theoretical framework

Step 6.

Compare and check consistency - are themes consistent, note differences, and ensure

interpretations are grounded in the data

Step 7.

Interpret findings - explain what the themes mean in relation to research question, existing frameworks (deductive part) and new insights (inductive part)
