# Supplementary material for: Barriers to accessing paediatric critical care and surgery: A qualitative study
Source: PLoS One. 2026 Aug 4;21(8):e0355278. doi: 10.1371/journal.pone.0355278 (PMC13436771; doi:10.1371/journal.pone.0355278)
Supplement: Supplementary file 6 [file pone.0355278.s006.docx]

**S6 Table 1.** Deductive–inductive coding framework.

| Participant group | Theme | Code |
| --- | --- | --- |
|  |  |  |
| 1. Children & Young People | *Access* | - Extremely worried about the pain but difficult to find the right words to describe it  - Needs the understanding, confidence and skills to ask basic questions about what is happening to them  - Paramedics were helpful and caring |
|  | *Design/system* | - Were not asked their view on clinical decision-making  - Noticed loads of poorly people in ED, overcrowded in corridors |
|  | *Communication* | - Child's expression of pain and clinical interpretation  - Perceived some hospital staff as not always listening  - Printed information provision was understandable to child  - Difficulty remembering what happened as was asleep  - Child and caregiver were never told what caused seizure, or provided with information or follow-up from hospital |
|  | *Improvements* | - Access to paediatric hospital staff who can explain to child what has happened  - Information on medical condition and what to expect so they can be prepared and recognise the signs  - Advance notice of surgery so child can mentally prepare  - Reduce worry and consider mental health implications for child patients |
|  |  |  |
| 2. Caregivers | *Access* | - Not knowing whether serious enough, difficulty recognising and understanding severity of child’s condition  - Uncertainty in clinical diagnosis, repeated visits  - Being offered telephone appointments when want to see a clinician face-to-face  - Queuing for 111 services and not being able to contact or access GP  - Difficulty in getting a referral through GP, as difficult to access GP in the first place  - Some GPs being slow  - 999 call blue lighted to hospital, lack of space in ED for child  - Positive experience with paramedics  - Geographic spread of services  - Discharged from ED without seeing a paediatric specialist and not provided with reassurance or further information, fearful for health of child following traumatic experience  - Lack of signposting, not knowing where to seek help  - Difficulty getting through the pathways to surgery - ended up via ED  - First went to GP followed by ED  - Frequent visits to ED and speaking with loads of different doctors  - Lack of transport to access to hospital, or to attend appointments  - Access to a car important  - Parking costs  - Experience in ED, not doing all the right diagnostic tests, 'having to know the process'  - Issues with limited space and overcrowding in ED  - Employment conditions and issues getting time off work to attend surgical appointments  - Others had supportive workplaces  - Difficulty finding childcare for other children  - Caregiver told by hospital staff there was no beds available on paediatric ward so stayed in ED  - Would pay for private healthcare due experiences if it was affordable  - Waiting lists  - Unexpected surgery, with little time to plan logistics and practicalities of hospital stays  - Financial, health and emotional impact/cost of hospital stays on caregivers  - Having to cancel plans such as holidays at short notice  - Referral was straightforward  - ‘Logistics and practicalities’  - Unclear pathways, difficult to understand processes  - Provided with instructions for day of surgery  - Stress caused by not knowing when surgery would be, can't plan  - Additional children with complex needs at home and having to stay on the ward  - Low-income and expected to afford expenses of hospital stay  - Younger siblings not allowed on ward |
|  | *Design/system* | - Impact on mother of not knowing what was happening  - Being given the wrong advice, told to ‘wait it out’  - Noticeable levels of understaffing and staff stress  - Waiting times too long  - Delays and cancellations mean medical problems of child are not being dealt with, subsequently affecting health of wider family  - Caregivers not offered refreshments in hospital  - Mentions unfairness in relation to accessing surgery  - Discharged without explanation of how to use specialist equipment for child  - Supportive consultants  - Uncertainty around dates and times of surgery appointments  - Impact of missed and cancelled appointments, falling behind in care  - Draining and exhausting for caregivers |
|  | *Communication* | - Caregiver’s conviction and belief in child’s pain when initially dismissed by hospital staff as minor  - Perceived some hospital staff as wanting them out of the hospital  - Caregiver advocating for child  - Caregiver encouraging child to question hospital staff and self-advocate  - Some medical staff not very forthcoming in explaining procedures  - Indecision between different medical staff  - Communication style of clinicians described as 'textbook'  - Difficulty in adults interpreting a child’s pain and judging pain thresholds as different for individual children  - Was provided with clear instruction on where they needed to be at the hospital  - Communication difficult due to busyness of staff  - Some caring and compassionate hospital staff  - Felt like they did not speak with a paediatric specialist  - Discharged without explanation or follow-up  - Not provided with printed follow-up information  - Service fatigue from having to explain to different hospital staff  - Feel let down by hospitals  - Received printed general information pack but no information on surgery and aftercare  - Caregivers having to chase follow-up appointments  - Fast turnover in surgery, some caregivers going in knowing what they need to ask, and understand system  - Caregivers need to know how to escalate if there is a system  - Receiving calls from hospital on weekend evenings about surgical appointments for child next day  - Not enough time for CYP and caregivers to ask questions  - Follow-up letters with aftercare not being sent out  - No follow-up appointment despite expecting one  - Lack of communication with caregivers during prolonged surgery  - Not informed on what will be needed after surgery e.g. catheter - nursery could not accommodate catheter care  - Unable to confirm dates for surgery  - Felt able to ask questions  - Staff categorising caregivers as 'visitors' though expecting them to stay on the ward  - Mixed messages from staff on visiting times  - Repeatedly asking staff for things to be done |
|  | *Improvements* | - Access to paediatric doctors and nurses  - Hospital staff explain the risks and outcomes of specific surgeries to child patients and families in accessible ways  - Offer CPR/life support/catheter training for caregivers  - Peer support groups for caregivers in similar situations  - Joint decision-making between child, parent and clinicians  - Caregivers sitting in on handovers  - Printed and accessible information for caregivers so they know what to do during pre- and during surgery e.g. where to wait, length of time  - Mentions VCSE as providing equipment and staff training on bereavement and plays an important role in mitigating barriers to access  - Provide mental health support for families  - Provide advanced notice of surgery so families can plan/prepare  - Provide caregivers with updates on waiting list position  - Baseline indication of time for surgery so can plan |
| 3. Healthcare staff | *Access* | - Contingent on geography, postcode and location of DGH  - Difficulty understanding and navigating systems for patients and families  - Multiple transfers between hospitals  - GPs as missing the signs and symptoms, not having expertise  - Economic determinants - hospital taxis and accommodation, emergency crisis funds available if needed, and chaplaincy offers funds if requested  - Financial pressure on caregivers from missing work  - Impact on families when local hospital services unable to provide specialist care  - Staff in DGH missing signs and symptoms, not familiar with care pathways  - Staff lose valuable and longstanding relationships with local families when specialist services are changed  - Lack of additional provision/uplift in beds in tertiary centres, lack of beds to meet need  - Lack of commissioned hospital transport to transfer level two patients to underutilised specialist services  - Certain cultural and religious events and practices as delaying access to care  - Employment conditions and lack of access to childcare for caregivers  - Caregivers not realising or recognising how sick their children are before they present at hospital  - Some children present at hospital were in a poor state of health before they even got there  - Long waiting times  - Increased admission during winter and not enough beds  - Fears around safeguarding and social services  - Needs outstripping demand  - Not enough staff for beds available  - Impact of COVID on waiting lists  - Caregivers and patients with learning difficulties  - Reluctance to access DGH as unhelpful  - Cancelled dental surgeries as no space to accommodate a child with complex needs  - Longer waiting times for CYP with complex medical needs  - Different referral criteria for dental services across region  - Different waiting times for dental surgery across region  - Insufficient physical space in hospital  - Lack of commissioned dental services across region  - Patient anxiety about dental surgery  - Prioritisation of adult dental surgery on mixed adult/child waiting lists  - Waiting times based on postcodes as inequality  - Increased waiting times for child with complex needs as inequality  - Delayed dental surgery increases risk for ICU admission |
|  | *Design/system* | - Different systems and ways of working over two decades  - Geographic remoteness of some hospitals  - Less access to paediatric medical specialists at local hospitals  - Lack of funding/underinvestment in certain areas  - Postcode lottery for where a child will receive care  - Adult ITU providing care for children  - Questions about resource allocation at a regional level  - Questions about financial sustainability of Level 2 in remote area  - Lack of beds, space and provision, units too small to match demand  - A gap exists for a child who is too ill for general paediatric ward but is not ill enough for ICU  - Difficulty attracting staff to work in remote region  - Staff rotation  - Not enough time spent in hospital to recover  - Multiple specialties involved in care for complex medical conditions  - Multiple transfers between hospitals  - All networks affected by distance and transfer times  - Age thresholds, what is a child and how is this defined in medical terms  - Questions 'child-friendly unit' when intensive care unit designed for adults  - Focus on physical health whilst there is an impact on all areas of life/health  - Lack of paediatric surgeons in remote hospitals  - Lack of specialist provision and staff  - Feels letting children down as network  - Concerns about safety and risk  - Distress caused by transfer  - Staffing pressures and inadequate staffing levels to meet needs  - Understaffing resulting in staff exhaustion and burnout  - Stress for staff being unable to provide specialist care  - Limited time available to build relationships with families  - Limited physical space for caregivers  - Food vouchers for hospital canteen provided to some caregivers  - Lack of mental health trained workers to offer caregivers and families support, with long waiting lists  - High thresholds and high need/demand for family support and safeguarding concerns in social care  - Professional and personal impact of service changes on healthcare staff  - A need to recognise and address the financial strain that the physical illness of a child has on family  - On the floor staff not asked views on restructuring and needs  - Impact on staff of service restructuring  - Impact on surgery if acute activity is high  - Loss of familiarity of DGH with move to tertiary centres  - Lack of shared records and data between hospital and social services  - Poor communication with social services  - Working in silos  - Misperceptions about dental surgery and the challenges it faces by other medical specialties  - Not enough paediatric dental specialists in region  - Shortage of beds for dental surgery  - Understaffing of anaesthetists or recovery staff for dental surgery  - Waiting lists for dental surgery  - Dental surgery perceived as less important |
|  | *Communication* | - Importance of providing factual, accurate, honest and compassionate conversations with patients and families  - Family involvement in clinical decision-making  - Importance of respecting the wishes of families  - Takes more time to sit down and explain something in depth in an accessible way to caregivers  - Agreements between specialists on certain conditions as 'straightforward'  - Thresholds for hospital transfer  - Disagreement between hospitals and specialists as frustrating and delaying care  - Difficulties communicating with transfer team, struggles to set-up transfer  - Reluctance for hospitals to collect/transfer children  - Feeling unsupported sometimes  - Concerns about understanding and informed consent process  - Difficulty for families, and for staff having to explain to families, when a transfer has been cancelled  - Relationships with ‘long stay’ families who have children with long-term disabilities  - Mechanism for feedback from patients and families in place  - Handover between medics  - Clinical leaflets on medical condition are in place for caregivers, unclear whether this information is shared  - Important to create conditions for patients and families to feel comfortable asking hospital staff questions  - Difficult to tell/know if the caregivers have fully understood, suspects sometimes they have not  - Lack of social workers and safe-guarding |
|  | *Improvements* | - Improve public understanding of the system and how it works through engaging information campaigns  - Invite caregivers to take notes and ask questions when something is unclear  - Ask follow-up questions to verify whether caregivers understand  - Nursing staff checking to make sure caregivers understand  - Staff recognising when caregivers are financially struggling  - Display information about financial support available for those on low-incomes during their stay at hospital  - All regional system working together  - Local Level 2 Unit in rural centres  - Standalone paediatric urgent care centre  - Long-term strategy is needed  - More advanced critical care practitioners  - Senior nurses mentoring registrars  - DGH to have capacity and capabilities to serve local population alongside specialist services  - Create and fund more specialist paediatric staff positions  - One nurse to every two children  - MH support for caregivers  - More safety-netting conversations  - Increased physical space and privacy on unit for caregivers  - Nurse-led care  - More general paediatric nurses  - Recognition and realisation from other NE hospitals that some hospitals do not have the right resources to care for critically ill children  - Information provision in the form of printed materials  - 24-hour access to interpreters  - Creating shared data systems with social services  - Provide hospital staff with training on cultural and religious practices that are relevant to care delivery  - Health prevention/promotion interventions to help caregivers recognise early signs and symptoms of condition  - Empowering caregivers through knowledge and skills provision  - Increase awareness of funding from hospital chaplaincy for caregivers on low incomes  - Staff asking if caregivers would like financial assistance (because of stigma they might not mention it)  - Medical mediation training for staff  - Trauma-informed mental health training on impact of intensive care for hospital staff  - Training on recognising signs and symptoms for caregivers via school nurses and health visitors  - More funding and more training and education on paediatric intensive care and surgery  - Mapping of dental services across region  - Redistribution of specialist dental services across region  - Regional oversight for referral pathways for dentistry  - Prioritisation of dental surgery as linked to several outcomes  - Improving dental data quality and systems  - Allowing CYP to visit wards prior to dental surgery to overcome fears and anxieties  - More waiting lists available for dental surgery |
|  |  |  |
| 4. VCSE & Community | *Access* | - Caregivers need time to prepare mentally for what is coming  - Cost of public transport  - Cost of purchasing specialist equipment for home  - Cost of running electricity for specialist equipment at home  - Physical issues reaching hospital without necessary equipment  - Gaps in statutory funding for specialist equipment, Trusts and LAs not funding specialist equipment  - Delays in funding specialist equipment  - Lack of specialist equipment to access schools and hospitals  - Health and educational inequalities in equipment provision  - Lack of trust toward hospitals  - Parking costs  - Repeated hospital attendance due to cancelled surgeries, caregivers experiencing poverty will have to pay for this plus childcare, depending on their situation  - Parent as a live-in hospital carer 24 hours a day  - Caregiver exhaustion, job/career affected  - Upheaval can happen anytime, need to be prepared at very short notice |
|  | *Design/system* | - Lack of disability awareness  - Different practices between hospitals  - Fears around lack of one-to-one staffing  - Cost of food at hospital for families living in hospital with child  - Different clinical disciplines involved with different approaches and views  - ‘Fighting a battle’  - Lack of beds or sleeping facilities for caregivers living with child in hospital  - Lack of consistent staff with whom trust can be built  - Lack of strategic commissioning and funding  - Delayed or cancelled surgery means that secondary health problems become primary concerns  - Being in the 'system' for so long  - Families go through multiple complex surgeries, never gets any easier  - Lack of equipment provision at home when discharged |
|  | *Communication* | - Emotion versus logical  - Fear of leaving child's side whilst on the unit  - Caregivers having to advocate for child and family  - Caregivers need the energy, confidence and skills to advocate for themselves  - Questions why caregivers being asked to leave room in which child is  - Caregivers excluded from medical decisions  - Lack of accessible and child-friendly information provision, especially for complex procedures  - Caregivers having to explain complex surgical procedures, risks and benefits to child  - Caregivers signing consent form when not feeling fully informed  - Caregivers not knowing the staff or understanding medical procedures and protocols as affecting trust  - Lack of reciprocity between families and hospital staff  - Problems communicating with some hospital staff  - Perceived moral judgements about the behaviour and feelings of some hospital staff towards caregivers (e.g. 'being strong') during traumatic experience  - Some nurses and doctors perceived as quite scary and intimidating  - Parents feeling unable to question surgeons  - Written communication with hospitals (having to chase)  - Families reporting negative experiences with registrars  - Fears around safety  - Admiration for clinical team over a number of years |
|  | *Improvements* | - Disability awareness training for hospital staff  - Mental health support for child going through critical care or surgery  - Mental health support on units for caregivers and signposting to mental health support  - Birth Reflections type service for children in PCC or SIC  - Consultants who explain to child and answer questions  - Play therapists and more intensive care nurses  - Caring for caregivers  - Empowerment caregivers  - Providing advocacy and care training to increase caregivers knowledge, skills and confidence to continue care at home  - Connecting with families diagnosed with similar conditions  - Building networks and communities  - Specialist equipment provision  - Training in specialist equipment for complex medical needs  - Facilitating access to trained PAs  - Helping families who have exhausted everything  - Law and legal services  - Providing confidence  - Rights-based approach |
