# Supplementary material for: Barriers to accessing paediatric critical care and surgery: A qualitative study
Source: PLoS One. 2026 Aug 4;21(8):e0355278. doi: 10.1371/journal.pone.0355278 (PMC13436771; doi:10.1371/journal.pone.0355278)
Supplement: Supplementary file 7 [file pone.0355278.s007.docx]

**Appendix 6**

**Table of illustrative extracts**

| **Theme/Notes** | **Illustrative extracts** |
| --- | --- |
| 1. **COMMUNICATION BARRIERS** | |
| **CYP’S DIFFICULTY COMMUNICATING WITH HOSPITAL STAFF**  Interpreting pain thresholds of children using medical tools  Mentally preparing for surgery which was unexpectedly brought forward and left the child feeling anxious ‐ child  describes being 'caught off‐guard'  Issue of not knowing what was happening ‐ not providing information on procedures would have minimised worry and panic  Difficult recalling what happened at hospital as wasn’t awake  YP never told what happened, nearly died and still unclear what happened, no understanding or attempt to explain to child. | “I felt like I was talking but they weren’t always listening to me as much as I would have liked them to” [ID05]  “I felt like they thought that because they’re the experts, we don’t need to have much input” [ID05]  “I think that the doctors were really nice to me, but I think they tried to be nice to hide the fact that they weren’t telling me much” [ID05]  “They just said to me, like, “We’re going to remove your appendix,” so I felt like I had to keep asking questions to know what I wanted to know”. [ID05]  “I felt like I had limited say” [ID05]  *YP’S view on advance notice of surgery –* “And also, I feel like they didn’t give me much advance, they just came in and said, “We’re going into surgery, let’s go.” [ID05]  “ID06: That was quite bad. That was a bit horrible, wasn’t it, because they said, “We’ll come and get you at lunchtime,” so we’re eating our breakfast, I was eating my toast and, “Come on, then.” “Come on then what?” “Come on, we’re going now.” I said, “Somebody told us it was midday,” and the surgeon went, “Oh, well, we’re doing it now.”  ID05: That caught me off guard.  ID06: I think that made him anxious.  ID05: So that I could mentally prepare myself”.  *Early discharge despite pain:*  “ID05: But then they did send me home, despite me wanting to stay in hospital for safety reasons, because of my stomach.  ID06: And they kind of downplayed your pain, didn’t they?  ID05: Yeah”.  “I think the paramedics helped the most” [ID14]  “I don't really know because I couldn't really remember anything. I just remember waking up, and there were people around. But when I got to the hospital, I was expecting cannulas in my arm, and every five to ten minutes. But I think it was like- I got there, and then I got put in the room, and then I fell asleep. And then, I woke up, and then it was like, they checked us one more time, and then I just went home” [ID14]  “We were thinking like, "Is it because I was doing football?" Because it was really cold, and maybe because I did a lot of insulin. So, that's what we were thinking, but we didn't actually get told what it was” [ID14]  “I think if we got told about that, you could have seizures when you're diabetic. Obviously, it would still be bad, but we wouldn't think as much of it.  ID13: Yes, I mean I didn't have a clue what was going on”.  “That's all. I just wanted- yes, that would have been it. You're diabetic, we'll give the diabetic team a ring, get them to give you a follow-up this week, just something, just to reassure me that I wasn't just going home, but it was just all over, just like that” [ID14]  *Child’s understanding of their medical condition from caregivers perspective*  “Yes, he knows. But even though he knows, even though he is five years old, he just understands us. He has got a maturity about understanding us. And he knows that he has some problems. When he goes to school, he just covers his full face, he covers his nose, mouth with a mask because we always tells him he might struggle otherwise; if he gets a cold, then he will struggle, so he always does like that. But moreover, we are getting a lot of support from the school, from the teachers, because whenever he goes out, they will make sure that his face, you know, his head, face, everything is covered. They will make sure that his jacket is worn properly” [ID08] |
| **CAREGIVER ADVOCATING FOR SELF AND CHILD**  Caregivers need to have the energy to advocate when completely exhausted | “Sometimes you are an observer as a parent. Certainly, a lot of our parents have come and said that, and if you’re not a type of person who speaks up for themselves or who will want to be involved, who needs to just check things first, you’re not going to have that confidence once you go home and if you don’t know those things. If you’ve never been in the system before, if you haven’t been educated on what you need to do when, then you don’t know that when you’ve gone” [ID03-04]  “If you don’t have parents that are able to advocate for themselves or have that understanding or have the English language skills, the literacy skills, the confidence of advocacy because nurses and doctors are quite scary and intimidating... There’s that ‘deference’ element” [ID03]  “He had an appendectomy. He’s talking about when we went in on the Wednesday, as an A&E patient, and then they advised him, the two consultants said, “It doesn’t look like appendicitis,” and they kind of said to me, like, “This could be a waste of time if we operate on him,” but I said, “Listen, go with your gut feeling, you're nearly 12, you know the pain,” because they were saying he was not showing as much pain as someone with appendicitis would usually show” [ID06]  “To be fair, I'm the sort of person who goes, “What’s happening now? What’s going on now?” Do you know what I mean? So, they probably didn’t have much choice” [ID06]  “We’ve always encouraged our children to ask questions and question things. I don't think that’s normal of many 12-year-olds. I don't think they would be that confident enough to necessarily question a surgeon” [ID06]  “If you don’t have parents that are able to advocate for themselves or have that understanding or have the English language skills, the literacy skills, the confidence of advocacy because nurses and doctors are quite scary and intimidating ...” [ID03-04]  Describes those who cannot advocate for themselves as ‘just being completely trampled’ and ‘completely overwhelmed’ [ID03-04]  “I think it's not understanding the system, and how it's meant to work. So, how can you advocate in a system where you don't know what's supposed to be happening? So, I feel like we've had to do a lot of advocacy for ******, and that's with us knowing the system. And if I'm totally honest, at times, I feel like we've abused our knowledge of that system because we know that things haven't been going how they should. And we've known how to escalate that, and we've escalated it, in ways that I'm sure members of the general public wouldn't.” [ID10] |
| **CAREGIVER QUESTIONING MEDICS**  Mentions fortunate to have access to North East children’s hospitals when other families have to travel to different parts of the country  Communication and answering difficult questions  Mentions noticeable differences in quality of service in different hospitals  Some feeling excluded from medical decisions and dismissed when asking questions – need confidence to question surgeons and medical decisions  Excluding/ignoring the views of father | “I’ve had a couple of disagreements with them, I’ve challenged them, and they have been reasonably okay. However, I went back up to the NICU ward, just to query a couple of things. Because at the end of the day, she was there with them for two weeks. I went up and I asked them a couple of questions and they were quite - 90% of the staff on that ward were fantastic, on the NICU ward. However, I got the one lady who was not really great with us up there, if that makes sense. And she said to me she can’t help, she didn’t know the full situation, the staff downstairs are really good, and she suggested I listen to them. When I got back down to the children’s ward, they’d already phoned the children’s ward, saying, “He’s been up asking questions.” And I was like, “Look, she was there for two weeks with them, I’m entitled to go and speak to them and ask their opinion”” [ID16]  “And it just felt like you were going behind- They assumed it felt like I was going behind their backs, somewhat. But that wasn’t the case. Two years ago, when COVID was on, me and my  wife had a miscarriage, he was 13 weeks utero when he stopped growing and breathing, heartbeat and whatever, I don't know even know how you- Deceased. Now I had an inkling that something wasn’t quite right, as a father. I phoned the hospital, I phoned the midwife, and said,  “Look, can we get [and ECG], because we want to check if they're in line with a pregnant woman at this stage. I said, “Look, can we scan her, because I’m concerned?” And to be honest, they said, “No, you're the dad,” basically, and everything else, and my concerns were just absolutely ignored.  And if she’d have been re-scanned after the three-month scan, they’d have found out reasonably quick. But unfortunately, she got to five months, before we found out. So for me, she was carrying a deceased baby for another two months after it passed away” [ID16]  “There was another consultant who said, “If we go in, we’re not taking the appendix out if it looks alright.” So we had before but I was, like, “No, I want him to go for the appendectomy, I've discussed it with my husband, and ******* as well, we’re going to have the appendectomy,” so the following day is where we’re up to now, isn’t it, so it wasn’t fine, so they took it out” [ID06]  “But I wonder whether he wasn't that forthcoming with information. I felt like I asked. Went in as like quite an educated parent, and went in and asked all the questions. So, I feel like- it's hard to know because you don't know if you didn't ask the question, whether it would ever have come up. But I feel like he hadn't told me that ****** would need a catheter for seven days. And I asked that question because I'd done quite a lot of reading about the surgery, and spoken to people before. Which was obviously quite huge if me and my husband had just taken two days. The operation day off, the day off afterwards, and then thought he could go back to nursery. And so, I feel like that was quite important to know about the surgery” [ID10]  “If they don’t want to answer the difficult questions, why on earth should someone with no medical training be expected to talk through...?” [ID03] |
| **CAREGIVERS’ DIFFICULTIES COMMUNICATING WITH HOSPITAL STAFF**  Difficulty in clinician’s gauging child’s level of pain  Some staff ‘talking at you’  Importance of communication via ongoing discussions with caregivers before and throughout (and afterwards/debrief which is currently missing)  An example of good practice with a consultant fully explaining what might happen, the risks and benefits to child, and answering questions honestly. Another where a caregiver [ID04] mentioned this helped them to feel empowered  Caregivers asked to leave the room during consultations and not being allowed to say goodbye properly prior to being intubated  Praised paramedics  Some hospital staff not understanding the trauma of events  Unclear instructions on visiting times  Treating caregivers as visitors, though expect caregivers to be there all the time without providing suitable conditions for them to be there all the time e.g. sustenance and accommodation, especially for low-income caregivers  Incorrect feeding plans from hospital | “And things that you're asking for to get done, don’t get done. Like they wanted to put her on some milk thickener yesterday, that request went off to the pharmacy early morning, and it didn’t come up until very late afternoon, just before the pharmacy was closing at 5pm. So it was a bit late, that should have been done a lot sooner […] She ended up having two bottles, I think, without the thickener in, because the medication wasn’t on the ward that she needed.” [ID16]  “Everything is really slow, decision-making is slower, communication is not great. But other than that, it’s okay. I think it’s just slow. Feedback is slow in respect of results, you’ve got to chase them for them” [ID16]  “When you're asking them some questions, like my daughter’s ammonia levels are up, and I’m worried about it. And they keep saying, “We’re not worried about it.” The normal rate should be below 50, she was 71 on admission. Two days ago, they checked it and it had risen to 82. They don’t know why it has risen, they said there could be multiple reasons why. They said, however, it could be sat in the tubes for a little bit longer than what it’s supposed to be, and that’s what has caused it to rise. And I’m like, “You're not giving me clear causes for this to go up, other than it could have been sat in the tube for a little bit longer,” if that makes sense. They're going to re-check it today, and if it’s gone up again, they're going to send it off to someone to investigate why. But that’s not the point, they need to be looking at it a little bit better. Because to me, she was born jaundiced, her ammonia could be related to her liver. And if they don’t work fast enough,  it could lead to a liver shutdown. Worst-case scenario obviously, but that’s an issue. And that was a hard thing. I knew something was wrong. And as a father, you're not the one carrying the baby, your opinion doesn’t matter. And it shouldn’t be like that […] It should be, “You know what, if dad is concerned, we’ll do something about it.” But it’s just a bit of a pain.” [ID16]  “I’m her dad, so it worked, I do have an opinion, I do have a right to ask for things to be done, and everything else. But otherwise, like pregnancy, stuff like that, it’s irrelevant what your opinion is.  But yes, generally they are really good. The majority of the time, they do listen, they do communicate, and they do sort of work with you. But I think, as a dad, that you have less right,  less challenge, less opinion, than what the mother would have” [ID16]  “I’d expect a lot more clear communication. Now in respect of my daughter, she was on the NICU ward, she was discharged from the NICU ward after two weeks. We were home for two weeks and she started having some sort of seizures. We took her back up, on the advice of the NICU ward, to the children’s unit. The conclusion came to why the seizures were happening was because we were overfeeding her. Now we’ve gone home on the feeding plan that they’ve given us, and they’ve already said that we were giving her way too much formula. And now I’m saying to them, “Why were we discharged on that plan? Why was it all cleared from a consultant?” And they can’t give us any answers. “And it’s you who have effectively caused her to have these seizures by the neglectful letting her go home.” If they’d have actually done their due diligence, and made sure she was going home on the correct amount of formula for her weight, there wouldn’t have been any issues” [ID16]  “She was sent to [name of hospital] and they wouldn’t even let me say goodbye to her or hold her. They were going to let me wave at her from the end of the corridor, and that was considered acceptable” [ID03]  “We know what diligent, conscientious, caring professionals they are, but I think for some of our families as well and wanting to question, I find, particularly, questioning surgeons and the clinical reasoning the hardest because I feel like there isn’t necessarily always that reciprocity in the decision making than maybe you would have with a paediatrician or a general medic, or even an anaesthetist, actually, I have to say” [ID04]  “We always tell them this is the problem, these are the underlying problems for our child, but everyone, they tell, I don’t know whether it is due to this, so for the time being, they just give him, you know, and it really gets us frustrated like that, without getting treated for the underlying disease” [ID08]  “It’s the ethos as well. When you are in critical care, because we’d been in there for long periods of time, you know what place it is, so you know life is lost in there as well. So when you have to go to critical care and when you have to go into surgery that you know is going to be followed with critical care, it’s being able to support and understand what a parent is going through and what family members are going through because people keep saying, “It will not happen.” But it already has happened” [ID03]  “We went back at three in the morning, didn’t we? And then that’s when they kept saying to you, “You're not showing as much pain,” but I said, “Well, he is, but he’s just got a high pain threshold.” They weren’t really downplaying him or anything, but it felt to me as well that they just wanted us to go out of the hospital and just wait, but then really we realised we were right, didn’t we, because they said it was really swollen and it could have burst” [ID06]  “It felt quite archaic, actually. I'm only 38 myself, so I'm not that old, but I felt like, you know in grandparents’ homes, it was almost like the doctor would just talk at you, and you didn’t need to be informed of what was going on, like I got that impression. I had to keep asking. I said, “Okay, so, this consultant said this and what…?” it was almost like, “We don’t need to tell you, we’ll just get on with doing what we’re doing,” sort of thing” [ID06]  “ID06: I tell you what I didn’t like, one thing I really didn’t like, and I was caught off guard. I knew I couldn’t go into the theatre. I knew that. No one told me that, though, but I just knew, because I’ve got four children. When I got there, the nurse said, “Don’t tell ******* that you're not going to be there.” Which I didn’t think that was fair, because when he woke up, he went, “Where’s my mum?” and they said, “We’ll get her.”  ID05: I said, “Why was she not there while it was happening?”  ID06: I don't think that’s right to do that.  ID05: And when they said that she wasn’t there, I felt worried that mum would be worried, and then as soon as she saw me, she was happy”.  “I think what they sometimes come across as, what I think needs to change a little bit, was they tend to go a bit textbook. They asked *******, for example, to jump, I think, at one point, and because he could, the doctor was like, “Oh, if you’ve got appendicitis you wouldn’t be able to do that.” But I think, well, you can’t just follow it like that, you’ve got to… Jumping up and down pain, I think because I'm quite a strong, heavy person, so I kind of push for it, I think if someone who maybe didn’t have that kind of personality, they might have just thought, “Oh, well, the doctor knows, I’ll just go home, do you know what I mean? Obviously, it wasn’t fatal, but something like that could be fatal, and if it wasn’t fatal it would have caused him more problems because it bursts and it causes an infection, you know, and you’ve got a longer time in the hospital haven’t you and that?” [ID06]  “Do you know what I think as well? I think what it is, they’re so used to doing all this, but they forget that it’s not normal for lay people and patients and the parents” [ID06]  **“**They kept saying to me, “Oh, well we don’t need to take it out,” then obviously they kept saying that to me. But, no, I understand what you're saying, and we discussed it, and we both had a gut feeling that there was something wrong. But I think the impression I got from the consultant surgeons was, “It’s not a problem and we’re not taking it out”, it looks alright, do you know what I mean? And it did come out. So, you know” [ID06]  “ID06: That’s the bit that I don't think is. Don’t get me wrong, they weren’t really mean, the doctors weren’t horrible, but it’s just a bit that I find, I kept asking the questions about the anaesthetic, the anaesthesia person, what do you call someone who does the anaesthesia?  Interviewer: The anaesthetist.  ID06: Anaesthesia, he was probably the best doctor because he said, “This is what I’ll be doing, this is what I am going to do, these are the risks with it, because I have to tell you, don’t panic, but I have to tell you the risks.” So, the nurses and the anaesthetists, but actually some of the higher ups, like the surgeons that were going to do the surgery, they were not willing to say […] Yes. So he was amazing, absolutely amazing, everything that ******* asked he answered, he said, “Well, if that happens, that’s how we monitor your heart rate before you go in and after and during,” absolutely brilliant, but the surgeon did nothing like that, he just looked at me and when I asked he was like, “I know what I'm doing,” sort of thing, “and you don’t need to know.”  “To be honest, to actually speak to somebody who sounded like they were bothered for a start would have been nice. I get it is a very stressful job. But to be given some kind of guidance or something a little bit more than, “Well, just wait and see.” Be it, “If that temperature goes up again, go straight to A&E. If the temperature comes down, do this. Here is some advice on how to manage that temperature,” bearing in mind we had done all of the normal things as in a cold compress. We had done Calpol, like all the rest of it or actually, if 111 had a way to be able to get directly through to your GP or can make that appointment on your behalf or something like that particularly with, I'm not saying that anybody is more important than anybody else, but if they were able to have access. I don’t know if they actually do and this person just didn’t want to, or be able to put a referral through to somebody, or do you know, just something that is a little bit more that would have made you feel like you were actually being supported?” [ID07]  “I said, "Okay, that's good news, isn't it?" And they said, "Yes, no news is good news." But then, more time went by, and you try - you don't want to be a pain, and I get it that unless anything- if something went drastically wrong, we would have heard. But you were told two hours, and then it's two and a half, and then it was closer to three, and I mean, my husband was much calmer because he's a bit more chilled. But I was just- I just wanted to hear that he was okay […] And that scared me because I thought, "Well, I'd not seen any two parents on the ward all day." So, I really thought something's happened, and I just said, "Is ****** okay?" And she said, "Oh, I don't know. I haven't heard anything." And I thought, "What do you mean? Like, what is going on?" And I heard the other nurse who was more involved with us said, "Yes, he's fine. He's in recovery." And I thought, "Well, that's all I want to know." I just want to know that we haven't just both been called in.” [ID10]  “At the hospital [ED], I said to the nurse, "I've never been shown." And the nurse, it sounds  awful, but I think unless you specialise in diabetes, I don't think a lot of the nurses are quite aware of it. She said, "What do you mean?" And I said, "Ah, well, they said, last night, the paramedics mentioned glucagon training." And she says, "Oh, well you can speak to GP." And literally, just like that, we were sent home. And I mean I know the nurses maybe didn't understand the trauma of what had actually happened at home. But I was like, "Well, has nobody passed a note on to  understand what we're actually-?" I wanted to scream from the top of my lungs, "Do you understand what we're here for? I've just literally had to perform CPR. I had to give her  mouth-to-mouth. Why are we going home?" But just like that, we were sent home. And then I had to ring the diabetic nurse. ID14 has a diabetic team but we don't see them unless we've got a date in clinic. Because again, they're so short-staffed.” [ID13_14]  “I just wanted somebody to tell me, "It was going to be okay." And it was just a case of, "Right, okay. Her blood sugars are back in range. See you later." I just wanted somebody to go, "You know what? This is how it is. You might go home, she might have another one, she might never have another one. But there's support available. And this is a number to ring." There was just nothing. We were just literally sent home” [ID13_14]  “ID14: I think I was expecting to have a cannula in my arm, and stuff, as well.  ID13: Yes, they didn't monitor her. So, they didn't- they [ED] were only monitoring her blood sugars through her own glucose sensor. And I was like, "Well, it's not always accurate." "Well, it's  running." I told them, "Yes, it's not always accurate. Sometimes, I prick her finger and her bloods are six, and the cannula- the sensor is she's saying ten." I just wanted somebody to just check her over. It was too busy”  “All five [paramedics] stayed, and then when we got to hospital, I could cry thinking about it. Three of them that stayed, when they went to leave were like, "Shall we have a group hug?" And we were all just standing, me and these three paramedics, sobbing. The girl paramedic was sobbing, and it was just like, "You are amazing." And I think we just thought- they could see how traumatic it was, they did everything they could. And it was just at the hospital, it was just like it was nothing” [ID13_14]  “Well, put it this way, in the weeks to follow, I looked into getting private healthcare because I just thought, "I can't do this again. I can't just be- if it happens again, I can't just be told to leave to  go home with her, after a few hours, after having to give her CPR." But I couldn't afford it. (Laughter) I work full-time, but it just wasn't affordable. And then, I calmed down, and was like, "You know what? Yes, we're lucky. The NHS is great." But then, actually, sometimes when I think about it, and think, "God, I feel really let down." But there's nothing really, we can do about it” [ID13_14]  “And staff actually being aware. Because while we’re on the children’s ward now, three different staff members gave us three different rules on visitors. And it’s just you don’t know whether you're coming or going with it, you don’t know if you're doing a bad thing and you don’t know if you're doing the correct thing” [ID16]  “The one thing that bugs me is they're classing parents as visitors. Now, for me, I’m not a visitor. They advised- Because I said to them, “Do we have to stay with her overnight?” and they were like, “You don’t, but if anything goes wrong, someone might not see it, if you're not here.” And I’m like, “To be honest, I’m not here to care for her, that’s your role, while she’s in your care.” They shouldn’t be relying on a parent to notify them of a child being in distress” [ID16] |
| **UNDERSTANDING & CONSENT**  YP’s fear of surgery, and difficulties for caregivers in explaining to their child what is going to happen to them and why, when they don’t fully understand themselves  Lack of training for caregivers on complex medical conditions following surgery e.g. tracheostomy care or positional support | ID04: “Think of all the negative stories that go around the circle of what is now our community. You’re still processing all those. You trust those people implicitly, but you don’t know who else is in that room. You don’t know the surgical staff other than surgeon and maybe the anaesthetist because they do come and introduce themselves, but often, you have the same anaesthetist. You can process that, but then you’re second-questioning yourself, thinking, “Well, who’s in charge of this bit and what happens if...?”  ID03: Yes, and you’re coupling that with not knowing the procedure inside and out and having that familiarity with it, and sometimes what can happen is, with the best will in the world from a surgical team in particular, it can feel quite dismissive for a lot of our parents as well. “Oh, well, it’s only this. Oh, I’ve done this 100 times.”  ID04: “And can you sign the consent form?”  ID03: “Just sign the consent form.”  “Yes I want to be able to explain to my daughter why it’s important because she’s like, “Oh, God, again.” [ID04]  “I feel when we’re talking about surgery, and we know that surgery is coming, it should be part of that conversation with that child so that parents aren’t put in that position where you’re actually attempting to drag your child out of the house because they’re having that emotional breakdown and you’re having to use your best actor face to not show that emotional breakdown because you’re feeling exactly the same’ … ‘Anyway, he’s asking loads of questions. He’s asking: Why am I going for this? He’s autistic as well. So it’s just that repetition, and what was sent out was just not...” [ID03-04]  “I had a conversation yesterday, I think it was around informed consent and people’s ability to interpret information that you're giving them. And I’d had a conversation with a dad with a little one, who had had their appendix out. And I was talking about how they hadn’t been to the toilet and how it slows their bowel down. And he said, “What are you talking about? They haven't had any surgery on their bowel, they’ve had their appendix out.” And I just thought, “Oh dear.” So I had to start right back at basics. And this was after the operation. And you think, “What actual informed consent has gone on in that scenario?” You know, they’ve obviously gone through all of the medical stuff, and felt that they’ve understood, but… And this dad has maybe not felt empowered enough to ask the right questions, or say that he doesn’t really understand. So there’s a lot of that, that I find is very evident, people don’t… You know, you’ll say to them- And again, all of these things are so multi-factorial, aren’t they?” [ID09]  “Or they don’t understand what medications they’ve had. “They’ve had Calpol.” “Have they had any ibuprofen?” “They’ve had some Calpol.” So they don’t know what they're giving their children.  And that’s difficult to gauge as well, because what expectations should we put on them, as medical professionals. Because again, we’ve got that knowledge, and that skews your ability to think, “Why don’t you know that? You should know that. You’ve given your child medicine, why don’t you know what you’ve given them?” And taking that judgment out of it” [ID09]  “There’s just a lack of understanding about what’s normal within childhood illness, and then balancing that out with hype and statistics about sepsis and all of that sort of stuff. It’s really, really tricky to get that balance right. Such that, when people come to the front door, they're coming for the right reasons at the right time” [ID09]  “I still can’t sign a consent form. I still have to, 12 years later, let her dad sign the consent form. Although I’ve made the decision and I’m 100% certain it’s right, what she’s having done, I can’t put the pen to paper” [ID04]  “ID04: It tends to be that way now with my daughter [laughs] He said, “Well, I tell you what I’m going to do. We’ll plan. We’ll have a look down. We’ll do this, we’ll do that, but we’ll do all of those things and this is what could happen. It could come out, you could be right and it could be successful. It could come out and you could be very poorly very quickly and you’re going to end up in PICU. You’re going to end up in Intensive Care, where you might be there for quite some time. It could come out and stay out for a few hours. Your hole could close really, really quickly. Then, on a night-time when you go to bed, you might not be able to manage. You’re going to end up back in that ward and then you’re going to have to have surgery because we’re going to have to get another hole in there.” He explained it all to her, and I went home feeling like I was empowered, she was empowered and I wasn’t then processing, all the journey home, “How am I going to put her feet back on the floor?” They’ll just engage with the child and tell the child all the good bits so that child leaves hospital…  ID03: So it’s not informed consent? Yes.  ID04: ... and you’re having to then bring that child back down and say, “Actually, this is what we need to talk about, and actually this is what’s going to have to happen.” Whether we’re doing that with social stories, with PECS, with pictures, with just constant conversations, with videos, it ends up as our job, and I think there is definitely something to... Because we have to take our child to surgery when our child is going, “No, I don’t want it. I don’t want it. I don’t want it.” I know I’ve got cardiac surgery on the horizon coming closer for my daughter and, literally, she’s quartered outside of our... I mean, she’s got issues that you can see. If she were to undress, she looks like a patchwork quilt. You have to prepare yourself to go over those things and explain what this scar is, what that scar is, what the other scar is. “Right, am I done now?” “No, you’re not.” |
| **HOSPITAL STAFF APPROACH TO COMMUNICATING WITH CAREGIVERS**  Trust  Anaesthetist communication style described as brilliant and absolutely amazing ‐ child felt well‐informed, and  understood that bit  Clinical communication described as 'textbook' ‐ i.e. dismissing pain of child if the child can jump on the spot  Nurse asked mother not to tell son that she wasn't going to be there during the surgery, which the mother didn't think was fair. The first thing the child did when waking up after surgery was to ask for his mother. When she wasn't there, the child started to worry, worsened by the effects of the anaesthesia  Participant mentions that surgery is not an everyday 'normal' experience for everyone, people are not used to it in the same way as the clinicians, and more explanation should be provided so patients and families know what is  happening  Described as a 'whirlwind' and not enough time to ask questions | “I think you have that insight that parents maybe don’t, because they just come and trust. And they have to do that, they have to be able to trust the people that they're turning up to, with their most precious thing in the world. And that’s so important. And we didn’t want people to come and feel let down, that they didn’t have what they had before. So we did fight hard for it not to happen, and it still happened” [ID09]  “I’d only had intensivist experience when the surgeon asked us to leave the room. She was in her own room. “Leave the room because we’re going to have a ward round […] Why would I be leaving the room? She belongs to me. Why?” [ID04]  “It’s the ones that don’t do that that I almost feel sorry for, the ones that’ll just pretend they’ve understood what you’ve said. I quite often find that if I feel like I’ve been in that situation in the quiet room or after a discussion with parents/caregivers then I’ll say to them afterwards, “What did you take away from that? What do you understand from that conversation? Can you explain what did the doctor say to you and what do you think that means?” Just to make sure that they have actually grasped what’s been said. Because sometimes I think the hardest time is when people don’t admit that they don’t understand. But, yeah, I think everyone always has a little bit of go Google now don’t they and says, “Oh, so I’ve looked it up, this is what this means.” But yeah, it’s definitely getting better but it’s still a barrier, it’s still there” [ID11]  “A big thing on PICU that we - I don’t know if other people are doing it but I do all the time, I always ask the parents if they think of something just to write it down and say, “If you think of something you…” or, “If someone’s said something you don’t understand write it down and if I don’t know what it is I’ll go away and find someone who does.” [ID11]  “I was with her the whole time. Because they didn't do any further assessment in the hospital. They just checked her monitor for her glucose. And I said to them, "Do you not need to do any tests to determine why this has happened?" And she said, "It's just a seizure. They're quite common with diabetics." And I was like, "Just a seizure." I've just had to give her CPR. But then, I was thinking maybe this is just how it is. It wasn't like [ ___ 0:24:39]. I just thought, "Wow." I was clearly away with the fairies, if I'm honest. I was in no fit state, and nobody said, "Do you want to speak to anybody? Do you need any advice?" It was just like, "You can just go when you're ready, have you got a lift?" Like that” [ID13_14]  “And everybody is working to do the best that they can, I don’t doubt that for a minute. But sometimes you’ve got to make sure that you're listening and hearing, and not just hearing what you think you need to hear, but actually listening. But I think that’s a lesson we all need to take on, every so often, isn't it?” [ID09] |
| **COMMUNICATION WITH HOSPITAL**  Appointment letters never sent  Admin issues in follow‐up appointments not being booked, then clinic fills up and the care for the child falls  behind because of delays  Chasing appointment letters and dates  Surgery appointments cancelled last minute after long waits in hospital, they reiterate how secondary health issues become primary problems  Visiting times as different across wards | “My son is actually going for an ultrasound. He’s had a rogue lump pop up on his ribcage, near a surgical site. Yes, he’s been two-week-waited for an ultrasound, which I had to chase because the letter just hadn’t been sent out. So here's another issue: barriers to write... I say, “Make sure your letters get sent out and the letters are actually accurate. You should not be chasing.” I got a backdoor number because of my husband and even I got short shrift. “You shouldn’t be calling this number. This is for GPs only.” I was like, “Well, does my son have an appointment or not?” I’m quite good at managing people, and she came around. I was like, “It’s my son. I’m just so worried.” She was like, “Oh, it’s okay. Here’s the number for Ultrasound. This is the number you need.” So I’m like, “Well, one minute, you’re really annoyed that I called you and now you’re giving me somebody else’s number.” Anyway, he’s asking loads of questions. He’s asking: Why am I going for this? He’s autistic as well. So it’s just that repetition, and what was sent out was just not...” [ID03]  “My husband rang up and just explained the situation, that we were quite anxious now, and the secretary said, "Oh, he's already been listed, have you not had the letter?" So, they had booked him again, we just didn't know. So, we felt that was a bit frustrating because we were obviously quite stressed about it. And it was, I can't remember, like three weeks later, or something. Probably not- two weeks later, because it was in August. But it's just not much time to organise”. [ID10]  “Because we always knew that ****** would need more than one operation on this. That when we got discharged the nurses said, "We'll book you in to see the surgeon in clinic in about four months." Was what he'd asked for. And then, we never got an appointment through. So, eventually, my husband rang the secretary and there hadn't been an appointment gone in. And so, she's like, "Oh, well, there's nothing left now." And we're like, "Well-." And she offered us a telephone consultation” [ID10]  “So, I guess that's kind of interfered again, but that's again, an administrative issue, which I get happens but we just assumed that referral had gone through. We mentioned once we hadn't  got a letter, and they said, "Oh, you'll get a letter in the post, don't worry." You just get reassured. And so, obviously, you don't chase because you don't want to be that much of a pain. And then, there wasn't an appointment. And so, we have fallen a bit behind. And then once all the clinic appointments are full, all the clinic appointments are full” [ID10]  “My daughter has been on the main intensive care unit on the children’s ward downstairs, and that’s where she is currently. But the different wards have different rules on a lot of the same  subject, if that makes sense. So it’s not clear and precise throughout the different children’s departments. Like visiting friends and family within [ ___ 0:03:09] is quite confusing. Different departments have different rules on visitors. It should be, “Do you know what, okay, this is the  case, and this is across the whole hospital.” Rather than different wards having different rules, it’s a bit of a pain in the backside” [ID16] |
| **COMMUNICATION BETWEEN HOSPITALS/ ‘CLASHES’ IN CLINICAL DECISION-MAKING**  ‘Clashes’ in clinical decision-making and ‘gaps’ in systems and processes  Clinical management between hospitals  Agreement between medical specialties is straightforward for certain medical conditions like sepsis, however for those children who are less critically ill, it is less straightforward  Agreeing and finding appropriate paediatric surgical staff as causing more delays: clinicians agreeing on diagnosis, clinicians agreeing on transfer, doctors at central hospitals agreeing to transfer, waiting for specialist opinions | “The barrier then is people having a different point of view and having a different opinion about whether a child should be moved. You tend to have that clash between a paediatric intensive care team or a specialist team over in ********* saying, “Look, the child doesn’t need to come to us, we don’t think they’re quite at that level.” Us, as a general paediatric team in a hospital 70 miles feeling like, “We’re really not happy with this child being here.” It’s that gap in the middle” [ID01]  “Communication between teams, between both paediatric and surgical and between ***********, ******** and *********, it just hasn’t been as smooth as it should be. I think, over the years, that’s been more a frustration for the staff rather than any child that’s particularly come to harm. There is that potential there and I think that could work smoother” [ID01]  “We had a consultant neurodisability specialist brought in to gatekeep, basically, because there were that many different surgeons and different teams going, “I can fix that bit. I can fix that bit. Oh, I can fix that bit,” and they were all just clamouring. It took this paediatrician, a specialist neurodisability paediatrician to go, “Whoa, no, no, no, no, no. Where is the team around the child? Where is the team around this family? We are not pulling this child in 11 different...” The surgeons, once it was pointed out to them, were like, “Oh, yes, yes, very sensible. Yes, great, great, great,” but the silo element of it was mad” [ID03] |
| **LACK OF PATIENT INFORMATION PROVISION**  Inaccessible information provision for CYP  Children worry about surgery in the same way as anyone else ‐ they worry that mistakes might be made, so they need to be provided with information so that they understand what is happening to them  In some cases, no aftercare information provided to CYP and caregivers e.g. child needed catheter for seven days following surgery  Aftercare and information provision afterwards as good. The biggest issue was getting the date for surgery in the first place | “What they do is they will send out a photocopied info sheet that’s A4, that I think is meant to be accessible to children, but quite honestly, not” [ID03]  “My little boy had a catheter afterwards. So, he needed at least seven days, not at nursery. I guess that's another thing that nursery weren't happy to provide catheter care which I understand is a bit complicated, and they would have had to have lots of extra training. But for me and my husband, a quite hectic work life, we had to, at two weeks' notice, get quite a bit of time off work. So, yes, sorry, I'm blabbing. But yes, I think the logistics side of things for us was probably a more complicated barrier” [ID10]  “No, they gave me nothing, just *******. I wanted to know the idea of what was going on each time, because obviously for ******* as well, as he would be, as an adult would be, worried about… They weren’t forthcoming with it. Because obviously, as a mum, I sat there and said to them, “How long will he be in surgery for?” and he said, “About half an hour because I don't think I'm going to take the appendix out.” Then he was there for two hours. So, after one and a half hours my mother anxiety was hitting the roof, so off I went to speak to someone. And they were like, “Oh, no, don’t worry, they might have just…” which I don't think that’s very fair, is it, do you know what I mean, to do that” [ID06]  “No, never got any written information relevant to the surgery itself. We got the generic pack, your child's having an operation, and anaesthetics and things like that, like the generic pack. But never got anything in writing with regards to what the surgery was- about the surgery itself. We obviously will have had a clinic letter from that February. But I can't remember. I don't want to say for definite, but I can't remember there being much detail about the surgery within that, or what the aftercare would be like” [ID10]  “I think what they need to work on really is, you know the aftercare, when they go home, ******* had a lot of anxiety in the week. He’d be like, “I've got this pain, should I have that pain?” they need to give out more information for when people go home. A nurse did ring us after a week and she asked if the wounds were healing, that was lovely, that was fine, but rather than that, though, can’t they give out information of what to expect” [ID06]    “But no, I feel like the debrief was really good, and the planning was really clear after that. And the care was really good. We ended up back in A&E the next night because he had a bit of a hematoma, and the consultant wanted to see us the next day. And then because ******* was a bit more complicated, I think when we went in to get the catheter out, normally that's nurse-led, and the consultant booked in to be there for that. So, I think after that, I think from the surgery onwards, I think the care was exceptional. But it's just that getting the date, I guess” [ID10]  “Like the Glucagon pen, I've never ever been shown how to use one, and when I said that to our diabetic nurse that week, she went, "I know you were diagnosed in the lockdown, and everything was just up in the air” [ID13_14] |
| **CAREGIVERS & IMPACT/CONSEQUENCES OF MISSING WORK**  Missing work due to sleeplessness associated with child's condition | “It’s so difficult because our son is not sleeping properly, and we are not able to sleep properly. And that’s affecting our work schedules because if you’re not sleeping properly, you’re not able to go to work […] And we are so tired. And actually first, at the beginning, just raising his head on bigger pillows, it was okay for him, but right now it’s not at all possible for him, he is really struggling with his breathing problems.”. [ID08]  “But obviously, explained there was waiting lists with COVID. And I just said, "It would be really  helpful, me and my husband are both working professionals, really helpful to have some sort of ballpark of when that operation might be." And he couldn't offer any indication of that whatsoever which I can understand, they don't want to make false promises, but it just felt like it was then really difficult to plan life, and we always felt like, "Shall we book this? Shall we not?" And I had exams going on at work, and it was just always there, what was already going to be quite a stress- I mean, it was a fairly obviously routine elective operation he needed. But the stress of it was always there, with no idea. And then, I was just at work one day, in July, and got a phone call out of the blue from the secretary saying, "Oh, you've been listed for two weeks, today. And we'd booked to go away with family. Been booked for ages, and so, I kind of got quite upset, and explained this, and the secretary was like, "Oh, you can't do that date?" As if I was meant to have kept, indefinitely, the next- oh, I don't know how long later that was, the next six months open. So, I spoke to my husband. In the end, I rang back and said, "We'd committed money to our family, we didn't want to let them down. So, we just said we couldn't do it." [ID10]  “Even, you know, we’re having a really hard time because we are not getting any proper time, so we are totally mentally stressed. Because you know, by seeing our son a whole night, without getting breathing, it’s really very stressful on us. And even we have lost a lot of days in our jobs, we’re not able to concentrate on our work, it’s giving me a lot of mental stress. And we are waiting for a long period for us getting it, his adenoids, and getting sort of, it’s really very hard […] No, we haven't been referred for our stress so far because right now, for the past one week, I am not going to my work […] Because of that reason, I am not going; for 1½ weeks I am not going to my work because I am not able to sleep properly at night, I just wake up.” [ID08]  “When it comes to our working, we haven't got any support from the hospital. From our, I don’t know whether my, for my job for due to this problem, I think I’m afraid that I will lose my job, because for the past 1½ weeks I’m not going. This is actually because of this” [ID08]  “What you were saying, about not being able to go to work, before, that’s a major thing, when they turn up – “I need them better because I’ve got to be at work tomorrow and I’ve got nobody else to have them.” And that’s a major issue. That’s a big thing that we can’t medically solve, because there’s nothing- “Yes, your child is poorly, and they're not well and that’s awful, and we’ll help you as much as we can, but we’re not going to make them better in this next couple of hours that you're with us. Equally, we can’t solve the fact that you haven't got any childcare tomorrow, and you need to go to work, but you can’t because your little one is poorly” [ID09] |
| **NOT RECOGNISING/ UNDERSTANDING SEVERITY OF CHILD’S CONDITION & WHEN/HOW TO ACT**  Conversely, presenting to departments out of worry ‐ 'reverse barrier' ‐ attending when unnecessary  Learning difficulties and understanding signs and pathways  Social determinants and learning difficulties as worsening situation/ outcomes for child e.g. nutrition, delayed  care, recognising signs and symptoms, dental care, hygiene ‐ increasing susceptibility to illness/worse  outcomes | “From our point of view, I think it’s really education wise for parents not realising how sick their children are necessarily before they present at hospital seems to be quite a bit of a barrier at the moment” [ID11]  “You kind of felt like you didn’t really want to go and just turn up at A&E and sit and wait, because you feel like, “Oh, well, is it that important? Is it not that important?” I know that is one thing that parents have said to us” [ID07]  “I don't know about put off. I think sometimes I would almost find the opposite. I would find that particularly- Again, I noticed a big shift at [DGH 1]. And whether that was just historical, from it being such a very well-renowned department, and the access that people had to services there, but people would come without even any attempt at seeking primary care help. And educating them about that. It was almost like a reverse barrier” [ID09]  “So I think it’s almost like a reverse barrier, isn't it? That they’re attending when they shouldn’t be, because they don’t understand what they are capable of managing at home” [ID09]  “If they’ve got learning difficulties because I was talking about all the education and helping people but what if you’ve not got the mental capacity to be able to take any of that onboard. What if no matter how much I teach you I don’t know that if x, y and z happens to your child, you need to take them to hospital, how can you be supported to understand that? How can you be supported to, I don’t know, have that taught in a- I don’t know, I don’t want this to sound derogatory but brought down a level so that you understand basically what you need to do to take your child to hospital?” [ID12]  “I’m just thinking back, where a child has been born into a family say for example where the mum’s got learning difficulties but then they’ve had a child who’s got worse learning difficulties and then they’ve had a baby. And it’s that grandmother who’s already got learning difficulties  and the mum trying to look after a baby and not having those skills to understand things.  And a lot of the- when children come in sometimes it’s just because they haven’t done basic things that need to be done. The children might come in looking really dirty or their nappy  hasn’t been done a lot. They might have a simple thing wrong with them like bronchiolitis, which is simple to me because that’s what we do all the time in PICU. But they’ll come in and there’ll be these other associating factors which are making them a bit more poorly. They might not have had very good nutrition or they might have really terrible nappy rash, which doesn’t sound a lot but you need to be in intensive care for a reason then that’s going to affect everything. You might have poor oral hygiene as a baby. I know you don’t really brush a baby’s teeth but if you put them in the bath and doing all those little things it’s always going to help your outcomes because then you’re more probably susceptible to getting something like sepsis or something like that.” [ID12] |
| **LACK OF SUSTENANCE PROVIDED AT HOSPITAL FOR CAREGIVERS**  High cost of hospital food, especially for those on low-incomes who are staying at the hospital  ‘not healthy’  More support for caregivers who are staying at the hospital | “Some staff however are very good and give the parents one sandwich. But they would only give- Really, if mum was staying, they’d only give mum the sandwich. I asked last night for a sandwich, I was starving. And they said, “No, we can’t really give you anything.”” [ID16]  “They give us a tea and coffee facility, and that is it. They don’t give you any- Even patient food, they don’t give you any of that. On the NICU ward, they give you a meal voucher for £3. Both me and my wife are unemployed, so that meal voucher was an absolute Godsend. It didn’t cover the full cost, but it covered a good chunk of it. On the children’s ward, we get no help. And obviously, if we’re there all the time, you’ve got to pay for your meals. If you're there for a month, you're easily looking at £300 on hospital food, that isn't really the best” [ID16]  “They're expecting us to be there, but one of us can’t really go home, cook a meal and bring it back to warm up in a microwave. It’s not healthy” [ID16]  “The only thing they say is breastfeeding mums can have a meal, because they're breastfeeding. Now to me, it shouldn’t matter if you're breastfeeding or not […] At the end of the day, if they're wanting us to watch the child effectively, if you're going off the ward [ ___ 0:07:28], so it makes no difference whether you're going for food, you're going home for ___ or you're going home to sleep. If anything goes wrong, like they said, they might not catch it. And you can’t have both ways. You're sort of diminishing your responsibility somewhat and putting it on the parents, but not supporting the parents that way” [ID16]  “There's no- you don't get a tea or a coffee. If you want a tea or coffee, you're looking at Costa or Subway, or there's no food for parents, which I totally get. And I guess I could have made a  packed lunch, but I didn't. So, I guess cost is another issue […] If we didn't have our own car, I'm not sure how we would have got ******* back, post-op, at 7:30 at eight o'clock at night, or whatever we did” [ID10]  “As the NHS I know on PICU we provide meals for breastfeeding mummies but that’s it really. So if you’ve got, I don’t know, three kids at home so the other parent’s having to look after those three kids at home and you’re the parent who’s in the hospital you then have to worry about-  [accommodation] House is provided so you have somewhere to stay generally, we can usually manage that. But they don’t get anything like food vouchers or travel expenses, we always have to go to either the chaplaincy for an emergency crisis fund or social work” [ID11 – hospital staff]  “The child is fed. Your breastfeeding mother is fed. But that’s it” [ID03] |
| **SYSTEM SAFETY**  **BARRIERS - Risk is in transferring and the distance to the right facilities. Risk is in the delays in transferring, due to clinical disagreement on thresholds for transferring a child between different hospitals. This would not be an issue (risk wouldn't exist) if had local resources and trained paediatric docs locally (then there is the issue of recruiting medics to rural regions).** **For example, mentions city‐based hospitals being 'reluctant' to collect children (sometimes). For example, clinical management of child on a general paediatric ward due to lack of specialist resources and staff.**  “I would divide barriers into two. On one side, I think, there’re a lot of barriers that come from our side, essentially, so within the hospitals, within healthcare, how it’s set up in the region, and to do with the staff and the resources that deliver it. I also think that there’re some patient barriers that are social and financial” [ID15] | |
| 1. **SYSTEM BARRIERS** | |
| **UNCLEAR PATHWAYS [linked to communication]**  **Referrers unclear on local services/geographically closest**  **Different referral criteria across region for dental** | “Actually the thing is that when we call 111, or the GP first, we have to tell everything to one person. Again, we have to continue; someone will call us, then we have to repeat it again. Again, they record it and again someone will call us. Again, we will repeat it, repeat it. And if someone is not breathing properly, if someone needs help, it’s an immediate, emergency matter, really it’s a long time for two hours to three hours for getting an appointment, that’s really, really, you know, for struggling for breathing as well, and all, it’s really a very big time” [ID08]  “When we went to A&E, sometimes there will be a clinical practitioner, sometimes there will be a nursing practitioner. Sometimes, I think we have, they just, for the infection for that, the lung infection, just for the infection they just give us an antibiotic, so the infection can be subsided for some time. But when we ask them for them, they just tell us that’s not our thing, you have to get it from [tertiary centre], or you have to consult your GP, and get a referral to [tertiary centre], or something like that, we are not the right person to tell that” [ID08]  “From a work perspective and I suppose probably from a personal perspective, one of the things that some of our parents have said to us have been things like they’ve maybe not been clear what to do, or where they need to necessarily take their child. They’ve maybe called up 111, for example, and the support that has been there hasn’t necessarily directed them to the right place, either to go to their GP or to go to A&E or wherever. They just feel like there hasn’t necessarily been the right level of guidance of where to go and when to go there” [ID07]  “We were waiting for over 45 minutes and couldn’t get through to speak to anybody. We had called 111 and they were like, “Just make a GP appointment,” and we were like, “But we  physically can't. We physically can't get in touch with anybody.” We did eventually manage to get in touch with the GP, and they completely diagnosed him over the phone so he was never seen. It cleared up for a couple of weeks with the medication, and then about a month or so before he died it had come back. We had rung up the GP again and again, without seeing him, they just re-prescribed some medication for him. It was nothing to do with his cause of death, and it was something that actually when he had died and we were in A&E, we had to point that out to the nurses” [ID07 – VCSE with lived experience]  “It really is those more in‐between children where they're perhaps not sick enough to need paediatric intensive care but they're too sick to be on a general ward and that's the gap” [ID01]  “There's something about age and just on the cusp of what would be appropriate for our surgeons and what would be appropriate for paediatric surgeons. If you get kids around about five years of age, obviously very small ones just go to paediatric surgery but around five years of age, there's some discussion about where and who is the best person to be seeing this. A slight frustration that we've had over the years, and I've had it once or twice myself recently, is where, as a paediatrician with a lot of experience, I'm looking at a small child with, I think, a surgical problem or I think needs to go and see a paediatric surgeon and they're very keen for us to get an adult surgical opinion on our side so that you’ve then got a surgeon speaking to a surgeon. We've often then got middle grade surgical registrars with very little paediatric experience and it just seems, sometimes, to slow the whole thing down. Whereas you’ve got children who quite clearly, if they're going to need an operation, it’s not going to be done in ******** because we don’t have the set-up or the surgeons or the anaesthetists for children that young. I think for those where it’s quite clear that we’re not going to be able to do an operation in *******, we need to be moving them probably quicker and not having those delays” [ID01]  **“**That’s true from *******. From **********, they don’t have surgical provision in *********, really, so for children there… If you’ve got a 13-year-old in ********** with suspected appendicitis, they’ll be moved to ******** where the surgeons there see you and would operate. We have had instances over the years as well of children, the four and five-year-olds in **********, transferred to ******* to see the surgeons there who are then not going to do anything because they're too small and then transferred again to **********. That’s just silly. If you’ve got someone in ********** who potentially is going to need surgery then it would be a smoother transition straight to *********. There's really no point to them stopping in ******** in a hospital that’s not going to be doing that operation. **I think that whole pathway just needs ironing out**” [ID01]  “I think it is all around just the pathway, which needs some work internally, which we’re looking at but, also, just about communication. It just needs the various teams talking to each other because, when you take the step back, it’s clear what should have happened. Communication between teams, between both paediatric and surgical and between ***********, ******** and *********, it just hasn’t been as smooth as it should be. I think, over the years, that’s been more a frustration for the staff rather than any child that’s particularly come to harm. There is that potential there and I think that could work smoother” [ID01]  “My viewpoint of saying, “You may not have needed to come to ED today. Next time, try going to the urgent care centre,” or, “Have you tried your GP?” And if I had time, in that situation, I would ring the GP for them and say, “We’ve got a four-and-a-half hour wait, have you got any appointments? This little one is fine, from my point of view, they don’t need to be seen immediately, they’ve got relatively normal observations. Have you got an appointment this  afternoon?”. But that, in itself, is a time-consuming thing. If we could do that for everybody, we would change the culture, and people would understand that they can get access to their GP services and they can get access to urgent care or walk-in. But we just don’t have time, so we just rack them and stack them in the queue” [ID09] – *REFERRING TO GPs for less urgent patients, though no time to do that either*  “It’s not really even as straightforward as the site that the child is closest to because I think that there’re some issues with awareness of referral pathways for children and dental services in the region. That crosses into both primary dental care and medical referrers, they don’t know who the closest sites and services are so there’re children that are bypassing their closest service to get sent into ******** because, either, the referrer isn’t aware of the closest service or they have a referral preference to refer in because they think that it’s an easier service to get referred into as well” [ID15]  “Unfortunately, the dental services in the region, they all have different referral criteria so people will refer into the service that they think has the shortest waiting list and is the easiest to get patients accepted into. So that’s waiting lists” [ID15] |
| **DISTANCE/RURALITY**  Unable to access higher level care in rural areas  Distance and transfer times  - Lack of specialist staffing  - Surgery in children ‐ centralised in city  - Geography and transfer times and delays, need to be in the right place/unit in the event that the child deteriorates, which is not currently happening and puts the child at an immediate disadvantage  For example, ID01 mentions not being set‐up to deliver the care that is needed to children, and a delay of two hours for children to receive the best care, which means they have to transferred elsewhere in the region  Lack of provision (space) in remote hospitals who are sick but not sick enough to meet the thresholds for transfer  Age thresholds for children for certain surgeries  Views that certain children need access to the right critical care services, and the system is putting them at a disadvantage from not having access to these services  Worry and concerns for staff due to situations arising as a result of lack of specialist resource and staff ‐ don't always feel supported by other hospitals, lack of recognition of what remote hospitals are dealing with  Geographic distance to hospital, and transfers, as impacting clinical outcomes.  Distance to services and access to transport a problem for those on a low-income. | “The main barrier that we face and the main issue is purely distance and that’s true for *******. We’re a sister hospital in the same trust with ********** and that’s even truer there. Children where it may be appropriate to manage them on a general paediatric ward in ********* where you know, if there's deterioration, that services are very quickly on hand” [ID01]  “I know if there is a transfer, it leaves gaps behind and makes things harder from **********’s point of view but I think to be so far away from those services and then potentially delay those services coming and more definitive treatment from being implemented, does disadvantage the kids” [ID01]  “It's a remote distance from that more definitive care” [ID01]  “The main issue we have is distance and the fact that there are going to be delays because of that distance in getting children through. Really, these more HDU-level children, we've particularly had problems with children. There seems to be something about if a child’s ventilated or not. If they're ventilated, it’s straightforward and, if they're not, it isn’t always” [ID01]  “We had a child about two months ago who was desperately sick, diabetic ketoacidosis. It  was discussed extensively with the teams in ********** who were very happy for this child to be left in ******** and said they wouldn’t be doing anything different but it was at a level that’s way beyond what we should be managing safely on a paediatric ward. You could argue that, yes, they don’t actually need intensive care and they didn’t need ventilating and those aspects but, really, they were way too sick to be on a general ward and there just isn’t somewhere for these kids in between. The child did very well and didn’t come to harm and was stable in the end but we did have, I think, a good 36 or maybe 48 hours of this child needing a lot of input and causing a lot of worry for the staff at our end who felt that this child should not be on our ward” [ID01]  “It’s kids who, I think if they were in *********, probably would have been in HDU in ******** or even in the same building as the intensive care unit. If a child in our ward deteriorates, ****** is at least two hours away. We've certainly had kids where they’ve been very unstable by the time they’ve moved and probably should have been moved earlier. I think we do feel unsupported at times. That’s, I think, our main issue” [ID01]  “We’d need a realisation from central services in ********* and they would need the right resources for that to be able to accommodate these more in-between children. Now, I appreciate they’ve got their staffing pressures, they’ve got their bed pressures and they're not just looking after us, they're looking after a whole region so I think it is something that needs to be properly  resourced but I think there is a level of children that we shouldn’t be managing in DGH hospitals” [ID01]  “There’s been things like the Waiting List Initiative to try and sort that out. But certainly within PICU, which is my little bubble, my little area, I haven’t found any barriers of people accessing PICU or the care run PICU. It’s mainly the geographical thing of managing to access PICU because of where you live” [ID11]  “Other major barriers that I certainly noticed when I worked for [hospital transport service] was almost like a postcode lottery, so where you live to the care that you receive” [ID10]  “We are remote from ***** ****, so it is quite a journey to get to us, and even longer journey to get to [name of town], if things are happening […] **So in terms of critical care, we do sometimes struggle to get help, and it is not that people are not willing to give help, it is more about distance and regional specifics**” [ID02]  “So it is at least one hour journey from [name of city] to [name of city], and a good two hours to [name of town]. So if things are happening and we decide the child needs critical care for [name of town] - For a child in [name of town] it, literally, means at least four hours to actually get to a critical care unit which sometimes causes a lot of distress” [ID02]  “So it’s great that we now have [hospital transport service] that brings the kids. But obviously if you live over in [rural area] somewhere out in the sticks near [rural coastal town], you’re not going to get paediatric intensive care until you arrive at your local hospital, get seen there and then get brought to intensive care. So I guess there’s a barrier in that respect as well” [ID11]  “It’s a very small minority of kids that do need intensive care, but obviously if they did, you don’t  want them to have to go halfway round the world to a local hospital and then be checked out from that local hospital” [ID10]  “And [one part of region] is very, very tricky. And bless them, they have to get on with things a lot by themselves, purely for distance. So even that in itself is a massive physical barrier because wherever they go is a long way, and takes a long time. And the impact that that has on their outcomes is evident, in some of the data we’ve seen” [ID09]  “Patient-wise, we have had patients who have had difficulty with the travelling aspect. So, for general anaesthetic, they get told that they have to go home by private car or transport.  Especially when they, say, are situated in ***********, for example, and they have to come into ********, we’ve had parents who find that really difficult to logistically organise because they are either low-income and don’t have enough money, they can’t drive themselves either, or some social reasons. So they have anxieties about travelling, they find it very difficult. Because they don’t have services on their doorstep, they find it even harder to get into [urban city]. If that makes sense?” [ID15] |
| **TRANSFER PROBLEMS BETWEEN HOSPITALS CAUSING DELAYS/CLINICAL MANAGEMENT**  Lack of commissioned hospital transfer services for children and young people [check].  Beds available but lack of transport services to get child there.  Conditions for transfer –  i) whether a child is ventilated, ii) severity/acuity of condition as a threshold for transfer.  Waiting and delays as causing distress.  Under-commissioning of dental services across region. | “We had many conversations during the night with the [name of transport team], when we requested for them to come and transfer her. And they were like, “No, they are not coming until she is intubated and on a ventilator.” And then our ITU consultant said, “But I don’t feel confident to intubate her.” So this was going on all night and in the morning when the ITU staff changed to the daytime team, there was obviously a new person who came and said, “No, I am going to speak to [name of transport team]. So he rang [name of transport team] and he was like, “Look, this is the situation. I am not going to intubate this child, I want you to be here. I am happy to intubate under your supervision but I really need some expertise here from the paediatrician who is expertised [sic] in ITU.” So they finally came, but by the time they came, this child crashed, this child collapsed. So this child almost died and required a lot of medical support before they actually managed to set off for the transfer, because she was so unwell by the time, when all the discussions were going on, that this could have been prevented. This could have been prevented […] So yes. And that is because we don’t have people who have enough expertise in paediatric critical care here” [ID02]  “This particular child, this baby, the delay was basically caused by, like, the team from our hospital not wanting to incubate and ventilate without paediatric expertise. And the paediatric team not wanting to come until the child is on ventilator. And there was no understanding that we are just not confident enough to manage this child in an ITU setting, because she was very complex” [ID02]  “There are critically ill children who certainly need to be transferred and even in these cases we do struggle, sometimes, to get them to the right place” [ID02]  “And what they always want, in the conference call, is a lot of numbers, obviously. So I had done a respiratory rate, heart rate, blood pressure, saturation, etc. And the numbers for this baby were not too bad but the way you could see the development, and that is what we saw in the course of the night was that she was just getting exhausted, you know, she was just getting to the point when she would just stop breathing at some point and we could see, but that is something you actually know from the clinical expertise. And the numbers don’t always have to say that, “Actually, this is going in the wrong direction.” It is still just numbers. But because they kept saying, “Well, the numbers are actually not too bad,” it was a situation when I was like, “Well, we should probably make up some numbers to make them come or something, to do something to actually get some help” [ID02]  “To be honest, this is usually pretty straightforward because we usually deal with the [name of city hospital] surgeons and they usually say, “Yes, absolutely we admit the child.” So the only thing we need to do is to call [name of transport team] and say, “We need to get this child transferred,” or we sometimes just blue light them with our ambulance because they are not critically unwell, at that point, but obviously need some surgery as soon as possible. So that is probably easier to access surgical care” [ID02]  “There's been cases over the years where there's been criticism of kids being really poorly by the time they're picked up where services in ******** were unhappy that this kid was so unwell by that point where we've, actually, felt that we've been shouting for hours for someone to come and get them and that we felt they should have been moved earlier” [ID01]  “Yes again, [regional transport service], who is our regional transport service, offer an amazing service to those children, but they're not commissioned to do that […] it was that there was no  transport provision put in place. There was no assigned provision, to transport these children around the region into the beds that they needed to be in. Initially, that happened with [regional ambulance service], and they did the best job they could. But as they’ve got more and more stretched, and as they practically couldn’t transfer the children in the same way […] But like I say, they’ve stepped into the breach. Which is great. But because they're not commissioned to do it, it’s the first thing to get bumped, if there is a higher priority, naturally. So there are limitations to it” [ID09]  “Similarly, they're not commissioned to transfer level two patients. So the south of our patch is therefore less utilised. We have level two beds at [specialist centre] at [name of town], that we struggle to get patients into, who might be geographically from that area, because we can’t transfer them to there, there’s no commissioned transport to get them there” [ID09]  “Sometimes we get reports back of the [regional transport service] team getting there and the patient has changed clinically slightly and then they're not happy to take them. So then the team, in front of the family, have to have a conversation about, “The child isn't as stable as we initially thought,” or their clinical opinion differs to the base team’s clinical opinion, having looked after them for several hours at that point” [ID09] |
| **DATA SHARING**  Waiting lists | “I think this can help in the long-term because I think a lot of dental data is often unseen because our data isn’t recorded the same as medical specialties. Different community services record data differently in terms of how patients are entered into a waiting list, what they need on the waiting list, and then, ultimately, how many teeth they have taken out, what they’ve had done. So that means, from a future planning point of view, it’s really difficult to then gather the data you need to say, “Well how many lists do we need, how many children should we see on the list?” because  everybody’s data is different so it’s hard to compare” [ID15] |
| **GAPS IN STATUTORY EQUIPMENT PROVISION**  Lack of specialist equipment provision prior to and following admission.  Need access to specialist equipment following life‐changing surgery.  VCSE sector as plugging a gaps statutory equipment provision.  Cost of specialist equipment during a cost-of-living crisis.  Specialist bed support and sleep equipment not funded, and being told they have to ‘make do’.  Lack of specialist equipment (and training to use it) following major surgery means that i. families secondary health problems may become primary, ii. delayed discharge from hospital as no equipment at home, iii. cost of self‐funding equipment, iv) seeking support from VCSE organisations to help.  Families unable to afford specialist equipment, having to stay in hospital until they are able to be discharged home (further equipment required for school too so affects educational outcomes).  Local differences between which equipment gets funded and doesn’t.  Health inequalities ‐ lack of specialist equipment, reducing access to hospitals and schools, and other spaces. | “We are plugging a gap in this statutory provision. You are talking about basic human rights. We work really closely with therapists in the community and we know that they are fighting this uphill battle, so we stand in solidarity with the OTs, the PTs, the community nursing teams. We’re all saying the same things. There is not the strategic commissioning decisions to provide sufficient equipment in the home”.  “£6,000 to £7,000 for a bed, £3,000 for a walking frame, up to £15,000 for a powered wheelchair, up to £13,000 for an off-road wheelchair, for example”.  “All of those things are supplied electrically, so when we’re talking about where the country is in terms of fuel and everything else, you are timesing it by numbers that you could not consider. We have families whose children are on ventilators, that need fully electric beds, that have hoists in every room, that have specialist electric baths for moving and handling, who then have feeding pumps”, plus feeding pumps and charging wheelchairs.  “You’ve got children that have got scheduled or unscheduled surgery. Now, even if you’ve got scheduled surgery, we know of families who have got children with very complicated needs. It’s been known for a long time, but this surgery is going to mean they need a very, very specific piece of equipment at home. But there’s suddenly a gap in the statutory funding that’s not going to be provided at home. So then what happens? Does that child then not have that life-changing surgery and then get secondary health problems as a result of the primary issue not being addressed? Do they then have to go self-fund a piece of equipment? Do they then have to live in hospital until they’re able to be discharged home? This is just a really common ... Or do they come to a charity like ourselves for...?” [ID03-04]  “All the OTs in hospital told us that. Yet we were discharged home with no bed support, no sleep system, and they were just like, “Yes, sorry, that’s not funded in your local area, which is [name of NE city]. Now, if you lived in [name of NE city], you’d have gotten that funded, but no, you live in [name of NE city], so all we’re going to give you for this tiny, premature little baby is an outsized bed. You can’t have one of those anyway, but if we could, that’s what you would get.” So we just had to make do. Now, he got a bed funded by the [name of organisation]. It all got sorted. However, I’m thinking, “My *** is going to need some serious, serious surgery in the future. That’s unavoidable, but how does he then be kept safe in hospital without one-to-one nursing, not in a locked high-sided bed where he could cause harm to himself? Does that mean I’m going to have to move into hospital to live with him as a live-in carer, 24 hours a day, providing that care for him, which is what I had to do when he was a baby?” [ID03-04] |
| **STAFFING**  Continuity of care  Issue is not enough funded posts, rather than equipment and staff training/qualifications.  Small children require paediatric surgeons ‐ too small for adult surgeons (rural hospitals).  New staff, staff rotation, frequent changes - medical rotations for junior doctors, high turnover of staff in district general hospitals, non‐specialist and junior  meaning things might get missed, and they might not know the care pathways because they're new.  Too busy, no staff available to check, no paediatric specialists in ED.  Dental surgeries getting cancelled due to lack of anaesthetists and recovery staff.  Lack of dental paediatric specialists in region.  Not enough dental specialists to plan dental treatment, means worse outcomes when treatment planned by  a non‐specialist. | “It’s just different staff members, depending who is in. Even the same staff members have been in that have cared for her previously, and it’s different staff. For me, it doesn’t ensure continuity of care really. However, they are good, all of them are obviously roughly the same ability, but it would be nice to just have that one point of contact when they're in on nights, on days, whatever” [ID16]  “I think another barrier comes from staffing. So there’re dental staff involved in delivering care, there’re anaesthetists involved, and there’re also the resources of having beds in the area in the hospital that the service is getting delivered in. I don’t really often hear about lists getting cancelled because there’s no dentist. It’s more often that there’s no anaesthetist or recovery staff” [ID15]  “I think a lot of medical specialties and anaesthetists that haven’t had experience working in dental, also, just don’t really have an awareness of the challenges that the service faces and the challenges that our patients face. I think that might be part of the reason why we’re always… Well we perceive that we’re always the first to be cancelled, but we have had a lot of lists cancelled for one reason or another” [ID15]  “There’re also children that have teeth filled where the more appropriate thing to do would have been to take the tooth out because of the depth of decay, that has to do with who is making the decisions and the access to pre-operative radiographs as well. So, ideally, all children should be  assessed by a specialist, having a general anaesthetic, and that’s not coming from a point of view of specialists think that they treatment plan children better. It’s coming from some research that was done that has shown that children, treatment planned by a specialist, are less likely to need a general anaesthetic repeated. Also, some children avoid the need for a general anaesthetic entirely, if they’ve had a specialist assessment, because they have a treatment plan through other routes” [ID15]  “On a tangent to that as well, we have commissioning standards that say that children undergoing  general anaesthetic for dental treatment should have a specialist involved in their treatment plan. There are only specialists in [names dental hospital] and one community dental service. All other children, in the region, are being treatment planned by non-specialists. That might have implications on their treatment planning in terms of a high risk of re-general anaesthetic for surgery, so they might have more episodes of care than someone who is treatment planned by a  specialist as well. So they have an inequality of what staff see them for dental treatment” [ID15]  “The specialists that we have are all centred around [names dental hospital]. Sometimes that can be found in other regions but it is to do with how services are commissioned in the area. At present, the commissioners have not commissioned specialists, even for assessment clinics, in other regions. I think it is to do with what they see as value for money, they can get two senior dental officers for the price of one specialist, and I think that they don’t appreciate the other  benefits that a specialist can have to the service” [ID15]  “Our intensive care unit is an adult unit and, on the whole, wouldn’t take children so these kids who really are HDU verging on PICU, end up being managed on our ward in a way that I don’t think is best for the child. I don’t think we’re staffed appropriately for that and that’s caused real concerns and real problems over the years” [ID01]  “Generally, your job is supposed to be X, but actually you end up doing Y because three other people have not come in today and there is nobody else to cover that job. There are no extra staff, no bank staff to come in and be able to cover it. They are naturally feeling the pressure of there not being physically enough people to cope with whatever it is that is coming through the door” [ID07]  “It is not about the equipment or about the qualifications of staff because I believe staff can be trained. It is about staffing itself, so it is about not having enough staff to provide that care. So, for example, if we have a critically ill child who requires a lot of attention at night, I would have 2 nurses on the ward and if I have 10 children there and 1 nurse is completely bound to 1 child, that means the care of the other 9 children is basically impaired” [ID02]  “I think about the medical rotation and the medical training, so the doctors change every six months. I have a similar thing in PICU. So the new doctors start and like anyone, they don’t really know what they’re doing compared to what the doctors who’ve just left do. So I imagine if that happens in a district general hospital and there’s newer people there, they might not know how to access things either because they’ve just started” [ID12] |
| **NOT ENOUGH BEDS TO MEET DEMAND**  Not enough beds or staff, so child being put on incorrect ward.  Surgical beds impacted if demand on ICU is high.  Children being given beds on certain wards due to limited space, mentions increasing likelihood of mistakes.  Lack of physical space.  Lack of space to accommodate child with complex needs, meaning lists are cancelled to accommodate. | “ID03: Or they’re just not in the specific, dedicated ward. My son should have been a neuro baby because he had a shunt. He was put on the kidney ward because, actually, that was the only space.  ID04: Right, that was the only bed?  ID03: As a result, there were issues with his care with certain members of staff”.  “But again, there was no uplift in beds at [DGH 1] for the children who may have needed to be admitted from [DGH 4]. And similarly, because the [DGH 2] didn’t have any uplift in beds at [tertiary centre], they would then refer to [DGH 1] as well. So all of these children, coming from these relatively small geographical, needing to be admitted with various medical needs, be them short-lived, acute conditions or those longstanding complex children, and no extra provision provided for them. So that has been, I would say, the major barrier that I have come across. And certainly in terms of managing that for families, and also managing that operationally within the departments that I’ve worked, particularly in the EDs, where you're trying to match beds up with the amount of need.” [ID09]  “Certainly at the minute on the unit the cubicles we’re outgrowing our cubicles massively.  Everything that is neutropenic has to go in a cubicle, all the children that have these issues with infection, but then all the infectious children obviously have to go in cubicles as well and we just we don’t have the provision” [ID11]  “Everyone was like, “Wow, we’ve got all these beds” but number one, we don’t have the staff to staff the beds so even if we do have 12, 13, 14 PICU beds open, we definitely don’t have that many qualified staff to look after the patients” [ID11]  “Kids are needing intensive care, especially the complex needs kids. Every time they have a surgery there’s a bed, a provisional bed booked in PICU for them, they might not always need it but it always needs to be booked. And I think it’s just the problem that we’ve got, we’ve got much  more need and I think it’s happened a lot more quickly than anyone who developed the services thought it would” [ID11]  “So things like surgery, surgical activity, will be the first thing to be bumped, if acute activity is high and pressured. Or specialist surgery, that requires an intensive care bed after surgery will be bumped, if there isn't any intensive care. Because intensive care is full of the acuity from winter pressures or whatever, respiratory” [ID09]  “There’s also an issue with beds, if there’s a shortage of beds we feel like we get lists cancelled. Sometimes there’re strange things that happen, so I’ve had a whole list cancelled out in the  community because they had an emergency admission of a child with complex needs. They were quite violent and aggressive, and it was felt that the ward wasn’t then safe for other patients, so our full list got cancelled for that reason. Although that can’t be helped, that was a full list of patients that then got cancelled because they had nowhere else in the hospital that they could safely house this one child” [ID15] |
| **WAITING TIMES & LISTS**  If they are not helped or delayed, then they have resorted to other routes, such as providing steroid nasal  spray to their child without prescription. Resort to trying to help themselves as whole family is affected, but dangerous and risky at the same time.  Perception that dental lists are not prioritised and are cancelled for non-dental surgeries.  Talks about having to exaggerate symptoms to get an appointment  Lack of follow-up and responsiveness  Disheartening  Problems arising for anaesthetising medically complex dental patients – longer and yet more medically complex, does a longer wait result in worse clinical outcomes – health inequalities  Waiting times for dental surgery - more children admitted to intensive care because of dental infection and facial swellings | “From within our service, I think one of the main barriers is waiting times. Children, for dental surgery, will face up to an 18 week - It’s usually at least 18 weeks just for an assessment.  Then they get treatment planned and placed on a general anaesthetic waiting list. Depending whether they need just extractions or whether they need fillings and extractions or surgery, so minor oral surgery that would be, the waiting times are different. Also, they’re different depending on which service in the region they’re being seen in. So, in one community service, the waiting time is three or four times longer than a different community service, all in the same region. So there’s a massive inequality, there, relating to postcode really” [ID15]  “Unfortunately, the dental services in the region, they all have different referral criteria so people will refer into the service that they think has the shortest waiting list and is the easiest to get patients accepted into. So that’s waiting lists” [ID15]  “We perceive that lists are cancelled, the dental lists are cancelled, in preference to other medical specialities because it is seen that our priority categorisation isn’t as high and that, perhaps- I’m not sure if they also perceive that our waiting times aren’t as high but we do feel that we get cancelled over other specialties” [ID15]  “We have had issues with getting lists for medically complex patients. So when you have children that have got additional complexities with their medical history, so comorbidities, then not every anaesthetist will anaesthetise them. They have to, obviously, be seen in the safest place possible.  Out in the community, there are only a few anaesthetists that will agree to do them if they’re, say, ASA 2, and that’s only certain conditions. Otherwise, all ASA 2 and 3 individuals will come into [names dental hospital] and or be seen at [names dental hospital]. Their wait is over a year, compared to four to six months for ASA 1 individuals, just because they have additional complexities” [ID15]  “We have difficulty keeping it. We can’t leave them empty. Even though we have intentions of using them, if they’re left empty then the anaesthetic coordinator will reassign our anaesthetist for the list. So we have to make sure that there’re patients put on eight or ten weeks in advance so that our lists don’t get reallocated. I think, that is pressure on anaesthetic services but, again, it seems to be it’s a… We perceive it as that we are seen as a lower priority and that they always want our anaesthetist for other things” [ID15]  “So because these children, a lot of ASA 2 and definitely all ASA 3 children, have to be seen in the one site, and there’re a limited amount of lists for that site, the waiting list is at least twice as long as their healthy counterparts. That means that, during the time they’re waiting on that waiting list,  their oral health is deteriorating. Holes in the teeth are getting bigger. They may be suffering more episodes of dental pain, more episodes of dental infection, they may have to take more  courses of antibiotics to tide them over until they get their admission date. Unfortunately, they also struggle to access preventive treatment because, often, these children might have other  conditions, neurodiverse conditions or cognitive impairment, which means that they struggle to cooperate for preventive care and simple oral hygiene, daily oral hygiene, tooth brushing, so they are getting more disease while they wait for the initial disease to be treated” [ID15]  “Theatre access hasn’t fully recovered to our pre-COVID capacity, so we’re still not having as many lists and being able to see as many patients on the list as before, but the theatre access is better. What’s not better is the child’s ability to access primary care dentistry. There’re a lot of  dentists that are closing down because of backlogs to do with COVID. Your normal six-month checks are maybe taking nine months, a year, before a dentist is able to see you. Therefore, things are being diagnosed later on and disease is more advanced. So, before, where we’ve been able to perhaps do a filling, now we’re having to take more teeth out” [ID15]  “The anaesthetic lead sorts out the anaesthetic rotas, from what I’m led to believe. I think there’s a coordinator that does the recovery staff as well. I think dentistry is, perhaps, a bit more difficult to manage than other surgical specialties because we have quite a high turnover. So we would see eight to ten patients on a list if it’s just extractions. Compared to other surgical specialties, their op time might be longer. So, for an anaesthetic case, we have about a 15 to 20 minute turnaround. So our cases only take 20 minutes. Other specialties, it’s a bit longer” [ID15]  “Actually, the waiting period is really, you know, is a little bit annoying. And as far as where my child is concerned, I know the waiting period for my child for the last three months he has been struggling a lot, so without getting, everyone has a lot of rights, but the right to breathe is something, you know?” [ID08]  “Actually we are having, you know, [steroid nasal spray]; we got it from back from [country of birth]. Because once the consultation and everything is, when we ever book an appointment for the GP, it’s really, you know, we will not get a proper time, so by consulting a friend from [country of birth], and they told us to apply [steroid nasal spray]; you know, the nasal spray. So it had worked for him, and when we are going back to the GP, they told us that we don’t use these types of steroids to side” [ID08]  “But my son is not getting his proper weight, that’s the main thing what worries me a lot. But if it is an adult, or something, we can do some remedies, but if a child is not able to breathe properly, it is not able to express, you know, he doesn’t know what is happening, so it has to be sort of as early as possible, no?” [ID08]  “Basically, it’s a long waiting time, even if you’re telling other things. Because I am also from a medical background, so to be frank, to be honest and to be frank, I have seen, I have heard from a lot of people, saying that you have to tell a lot of more things if you want to get an appointment.  From being from a medical field, from a medical background, I don’t want to tell all those things, but my friends, my neighbours tell me if you want to get an appointment to [tertiary centre] or for A&E, if you want a quicker appointment, you have to exaggerate your illness, you know?” [ID08]  “Actually the things that we heard from our neighbours and friends is that to just exaggerate the illness, that will then lead you to get an immediate appointment. That has to be stopped, you know?” [ID08]  “When we were diagnosed, we were told, "You'll see somebody once every couple of months, and we'll come to the house, and we'll do some training for grandparents, friends." That never happened. We're still waiting now two years down the line. But in fairness, I've just become an expert myself, through groups on Facebook, YouTube tutorials. We were in the lockdown, I sat in the garden one day, for hours on end, researching it. Ordered some books from Amazon, and just taught myself everything that I needed to know. So, I've not really ever bothered with the hospitals or the doctors because I've just- like now, if her bloods are a bit out of sorts, I just adjust her ratio myself. Which you're meant to ring a nurse for, but nobody ever gets back to you, if I'm honest. But that night in the hospital was the one time I've really felt let down. I was like, "This is pretty crap." (Laughter) I thought she was dead just a matter of hours ago, and then now, we're just told to go home, just like that […] There was no follow-up. There was no- and then the doctor  did- I said to the doctor, "How do I go home and rest?" And he said, "Well, we can get you back in this week and do some-" I think they call it, what's it called? Something training, like- Basically, like CPR training but they called it life support training. So, we can get you some life support training. I'm still waiting for the call now. There was no follow-up from the hospital, there was no- there was just nothing. Our GP, I don't know if they're aware, but there was no call from the GP. I had to ring the diabetes team and say this is what's just happened. And the diabetes team, they cover ****** and **********, and they were the ones that got us down to ****** ** ******* Hospital that week, which is where we're living. Again, they were the ones that we talked through it all with, but then they said they would do some Glucagon training, but again, still waiting […] **But we've had to just get there ourselves. There's just been nothing. I've had more support from friends who are nurses and healthcare professionals, who have said if you ever need anything, and just like that, we were sent home.”** [ID13_14]  “After all of that, nothing. No follow-up with our own GPs. Nothing, absolutely nothing. I've rang the diabetic nurses myself, who said, "We'll do this training. We'll do that training." We'll get you down and do some Glucagon training." But we're still waiting for that as well. But nothing from the hospital, no follow-up, no check-up schedule, nothing, nothing at all” [ID13_14] |
| **HOSPITAL PHYSICAL SPACE**  Lack of disability awareness in the NHS  Sleeping facilities for caregivers at hospital.  Mentions that it is expected that caregivers will become live‐in carers 24/7 at hospital (and home) ‐ impact on them.  Caregivers expected to stay in the hospital 24‐7, though there aren't sleeping facilities. This may be particularly  uncomfortable for caregivers with pre‐existing health issues, or who are pregnant, as they mention.  Access to accommodation provided by charities, though considered lucky if allocated a room [flats shared with other families].  Staff try to help parents to rest by providing pillow, sheets and blankets, though there is limited space, and parents may not want to leave the side of the child anyway. Mentions unspoken rules of respecting the space of caregivers with a 'child on the edge' in critical care.  Access to small kitchen for some caregivers, however they also mentioned that they wouldn't have left the child to go and eat anyway, especially when they don't know the clinical team. | “It’s that disability awareness, isn’t it? That inherent ableism often pervades the medical profession. I hate saying it, but it’s so true” [ID03]  “Sometimes there’re strange things that happen, so I’ve had a whole list cancelled out in the  community because they had an emergency admission of a child with complex needs. They were quite violent and aggressive, and it was felt that the ward wasn’t then safe for other patients, so our full list got cancelled for that reason. Although that can’t be helped, that was a full list of patients that then got cancelled because they had nowhere else in the hospital that they could safely house this one child” [ID15]  So we were sat in the family room, she was on continuous monitoring. We know the continuous monitoring very well, with having two weeks up at NICU. But they pretty much use the  continuous monitoring as an excuse to put her back in her room, rather than have her sit in the family room. And I said, “Look, if there were any issues, I’d be telling you”, rather than confining us to a 3m x 3m box” [ID16]  “These are families who, if you have a child with a wheelchair, that uses a wheelchair on the whole, more than likely, they’re going to have through-floor hoists. More than likely, they’re going to need a through-floor lift. More than likely, they’re going to have to have a specialist bed. All of those things are supplied electrically, so when we’re talking about where the country is in terms of fuel and everything else, you are timesing it by numbers that you could not consider. We have families whose children are on ventilators, that need fully electric beds, that have hoists in every room, that have specialist electric baths for moving and handling, who then have feeding pumps” [ID04]  “So then you’ve got this for parents when they’re attending either planned admissions or acute admissions. How do they get all the equipment? How do they even get their child into your hospital knowing: Is their child going to be able to be adequately supported in hospital? Obviously, when it’s an emergency, you don’t have a lot of choice” [ID03]  ID04: The consideration around access is getting into a building.  ID03: It’s not just a ramp. Do you know what I mean?  “There isn't really anywhere to go, or then you step to the outside and you’ve got domestic situations going on, other people that are arguing about their child while you are in a really difficult position with yours. Even if there was just, I don’t know, a quiet room or a quiet  area, or something where somebody could direct you to, to be able to go and do whatever you needed to do. I think that would be a massive benefit” [ID07]  “So there were things like a dedicated breastfeeding room. There was a dedicated mental health room off the high acuity area, which was safe, ligature-free, all of that kind of thing. That had been a separate protected space. And don’t get me wrong, it would flex, if it wasn’t being used, it would flex into other things. But that was there. I think the biggest thing I found, certainly from an ED point of view, that I thought was an amazing investment, was the parent room next to the [resuscitation] room. So used for if a child had passed away or was very, very sick […] And I thought that was a really valuable investment, and something that, thankfully, didn’t get used very often, but when it did, was invaluable. Absolutely invaluable.” [ID09]  “On the wards, again spaces that are premium all the time. So I think they do have decent facilities for children. They’ve got a lovely big playroom next to the orthopaedic ward, that has got  staffed by nursery nurses and play specialists, which is great, and very well-used, especially for the day case children. And they would have pull-out beds for parents to stay in, I think they had a little parents’ kitchen on each ward, or certainly somewhere they could go and get a cold drink, if not a hot one. But again, they didn’t have, like [tertiary centre] have, single rooms, ‘cubiclised’ rooms, they were still bays, with parents in each bay” [ID09]  **“**I would often say to families, “This isn't because we don’t know how to look after you, this is because we’re not able to keep you for any more time.” And the physical space, certainly at [DGH 2], demonstrates that there is no space [...] So that is potentially, yes, a barrier, certainly for those children who have got complex needs. Because they aren’t getting that level of care anymore for that length of time that was previously offered. And does that have a knock-on effect that the skills that the staff are able to offer? Long term, yes, I’ve got no doubt it would do.” [ID09]  “It depends where you are. If you’re talking about critical care on PICU, no. A visceral, visceral memory is sleeping in the parent coffee room. Without fail, every week, there would be new parents, and the nurses would do their absolute best. They’d bring through a couple of little sheets and blankets because PICU, for obvious reasons, does not have parent accommodation as a possibility. It’s just not possible, but what they do is they accommodate the parents for as long as they possibly can during the day and the evening and then they’re very good at nudging you to either a parent room, where there are teas and coffees and you will always see parents just sleeping on the sofas... It was the unwritten rule, wasn’t it?” [ID03] “If there was a parent sleeping in that room, you just didn’t sit in there and have conversations. You would go to the [name of hospital restaurant] or you’d go somewhere else because that parent has a child on the edge. It was this unspoken thing. So the nurses would provide, and if it was really, really bad, you would sometimes be allowed to stay in the interview room. There’s a little consulting/interview room, and on those worst, worst nights, they would put in pillows, sheets and blankets and it would be, literally, maybe, what, a 20-metre walk on the ward. That’s it. That’s what there is for parents”. [ID03]  “We were very lucky because we stayed in a flat but it was a shared flat with two other families. It was the flats that they used to use for transplant patients when they were building up their living skills and they were no longer in use. Again, in exactly the same position, if a family had come from further afield, we would have had to go, but in peak traffic, I’m not getting to the [name of hospital] for over an hour. In decent traffic, I can make there it in about half an hour-ish”.  “Yes, the corridor [in ED] was seriously crazy. There were just people in the corridors, on beds, looking on death's door. It was scary. It wasn't a nice place to be. We went from a corridor, then  luckily, she did get actually put in the room, I think because she's a minor. But when I said to the woman, I said, "We're not on paeds here, are we?" She said, "No, there's no room." And I said, "Oh, right, okay." And she said, "But we're going to get you home soon anyway." And I pretty much said, "I don't really want to go home. I'm scared." And she said, "Oh, there's nothing to be scared about. She's had a seizure. They're quite common." So, I'm like, "Oh, right, yes, okay." And then, I got in the car, and I was just like, "What the hell's going on? What's just happened? Why are we going home?" It was just crazy, scary, crazy” [ID14] |
| **ADMISSIONS PLANNING FOR CHILDREN WITH COMPLEX NEEDS**  Severe anxiety about dental surgery  Neurodiversity  Carers    Lack of hospital support services  Disability  Transport issues  Challenging behaviour  Lack of side rooms on wards [physical space]  Longer waits for children with complex medical needs as not every anaesthetist will anaesthetize them, e.g.  have to wait over a year compared to 4‐6 months.  Inequalities for children with complex needs, worse clinical outcomes and longer waiting times making it worse, as more advanced disease. | “Something that we see really regularly is patients that have severe anxiety, or neurodiverse conditions, that find coming to the hospital very difficult. Often, there’re not enough support services around them to facilitate that so they, perhaps, need more staff to help safely treat them. Perhaps they could benefit from visiting the ward and seeing the facilities and being introduced to the members of staff who will look after them, and that is possible but often very difficult to  coordinate. Their care, then, gets deferred until all that can be teed up. Patients that need carers, so that come from residential settings, often need additional members of staff, need transport. If their transport is cancelled, or they don’t have enough staff from the residential setting to send the patient, then their care gets deferred as well. Yes, so I would just like to see additional support for admission planning, really, to do with them” [ID15]  “Some of our patients that are very complex, they need to go to pre-assessment clinic. I’ve had  patients have to wait three months for their pre-assessment clinic appointment because they only have certain designated slots that dental can book into. The fact that it has to be a consultant-led pre-assessment appointment, because of the complexity of the patient, means that it’s harder to access that clinic appointment so the patient is again… Their treatment is deferred three months until they get that appointment. Then they get planning, then we have to have a multi-disciplinary meeting about them. It all just pushes the admission date back, back, and back” [ID15]  “For some patients, who require side rooms as well. So if they’ve got neurodiverse conditions, if they have challenging behaviour, there’re only a limited amount of side rooms and often patients get. The request for a side room either goes overlooked or they don’t have enough side rooms to give them, and then the admission doesn’t go well because of that, and then the patient is cancelled because they are uncooperative and we can’t bring them round. So they have to be re-booked with a side room, first on the list, so they get their care delayed as well. We find a lot of anxious teenagers fall into that category. They don’t want to wait around. We’ve had teenagers abscond from the ward, and have to be brought back, because there hasn’t been enough preparation and planning in their admission because, I perceive that, those support services aren’t really available or, if they are available, they aren’t known to us” [ID15] |
| **TELEPHONE APPOINTMENTS RATHER THAN FACE-TO-FACE** | “But it’s a long time because after we went to the consultant for the two times, they are relying upon steroids, they know all that it’s a matter of urgency, but still now they have just given us a telephonic appointment. What, from your side, what do you think about that?” [ID08]  **“**Again, a telephonic appointment is not at all necessary, I think they have to see him; a face-to-face appointment is needed so that they can notice how much extra help [ ___0:17:54], how much he is suffering” [ID08]  “And again, it will be giving a lot of mental stress. And then we will be automatically as a common parent, we will be just thinking that okay what can be done with that telephonic appointment? Even me and my wife, we are talking like that; what can be done with a telephonic appointment? We are again recording, the GP has been recording and putting everything from ourselves, and just you are giving us a telephone appointment. And GP have told that sometimes this may be removed surgically, he needs a surgical removal of the adenoids, then again they have given just a telephonic appointment. And I don’t know what is the difference between ENT, to see ENT and getting referred from the GP, but they’ve just given us. And the GP has referred ENT with immediate effect, so they’ve just given us a telephonic appointment. It is a little bit worrying for us, and it’s really giving mental stress for us.” [ID08] |
| **PARKING COSTS**  Parking costs described as 'extortionate' ‐ have to pay for the full day if only staying for an hour or two. 20 mins parking for free though not enough time to get across hospital, especially when walking in pain ‐ then £6.50 for up to 8 hours. | “The parking is extortionate, and then I forgot, and I'm going to appeal it though, we got fined. See, I don’t drive, my husband had to take all the kids out, but the parking charges at the hospital are really extortionate. And then where my husband was coming back from the hospital to bring us up, he kept having to pay for different parking, the full amount, you have to pay for the full day, even if you're only staying for an hour, or two” [ID06]  “I think it’s unfair to charge… they give you 20 minutes for free, but, I'm not being funny, you can’t even get to the other side of the hospital in 20 minutes, do you know what I mean? Especially when he’s in pain walking slowly with his appendix and we’re trying to find a chair for him and that. But what I thought was quite bad was that it was like, every time it was like £6.50, but that gave you up to, I think it was up to eight hours, I’ll check that with my husband though, let me double check the amount of time” [ID06]  “Obviously, there's a cost to all- obviously, not a cost of the operation, but there's a cost of parking, it's ridiculous. I mean, we saw the surgeon last week, and both me and my husband wanted to go to that appointment. Which I'm not sure why because it was another three-minute appointment. But we were in and out, and I can't complain. Such quick appointments. We were seen before time, I think, it was really good. But our car parking was still £4 which isn't- that's fine. But for some people, that's not fine, is it? And it's a lot of money” [ID10] |
| **SYSTEM RESTRUCTURING**  Loss of familiarity for local people/populations.  Lack consistent and familiar hospital staff (trust). | “Then the shift was to centralise the services more to the tertiary centres […] But our concerns, as the ground shopfloor nurses, was where are our children going to go with complex needs, with longstanding health conditions? Because if they present to hospital, those parents present because they know they need to be admitted. And we know those children best, having looked after them for many, many years. They're not going to want to come to us, they're not going to want to come to their most local hospital because they know they're going to then need to be transferred” [ID09]  “Just go straight to the [tertiary centre], because they knew that that’s where they were ultimately going to end up. And why waste 24 hours and a transport, when they could just present there in the first place? So from a staff point of view, we found that really, really hard, because we really valued looking after those children. We understood those children, we understood those families. We knew the dynamics of the families. We knew that, when they came, they didn’t have to tell us the full story, because they were our families, and we had ownership of them. And took a  great pride in that” [ID09] |
| **PHYSICAL & ECONOMIC BARRIERS** | |
| **SOCIAL DETERMINANTS -POVERTY, CHILDCARE & COST-OF-LIVING**  Socio‐economic factors not figuring in medical decision‐making because the focus is on physical health ‐ does mention social determinants do come up sometimes relating to finances, and the cost of getting to the hospital and then issues around accommodation.  Caregivers move into hospital to live with children, 24 hours a day, when they have other children to look after too.  Cost of food for caregivers staying at hospital with child described as extortionate.  Caregivers stop work, careers affected, and pets have to live with other people.  Lack of joined up systems between hospital and social to understand social determinants  Lack of access to social work teams on weekends  Safety issues regarding background of some family members e.g. domestic abuse, sex offender register  Cost of transport to access hospital | “I have had patients that have not been able to afford the transport. They’re not entitled to have their transport paid for them, that makes it very difficult. One patient had to phone the council and get some of their rent money refunded to them so that they could pay for their child’s taxi after their general anaesthetic. It’s not very clear what financial support is available to these people. I think it probably is out there but it’s just really difficult to access. If information like that was more prominent, or awareness-raising around that, that would be really helpful” [ID15]  “More support for parents, to be fair. Clear communication. Meals included. If they're wanting you to be on the ward, they need to be supplying you with some sort of food, to be fair. Because you can’t give effective care if you're tired, if you're knackered, if you're starving. And you get more tired, you get lethargic, you get ratty. And when parents are unemployed and don’t have the money- Look at our region, it is one of the highest unemployment rates in the country” [ID16]  “That isn’t usually a factor in these kinds of decisions. We usually have a child who’s deteriorating and everybody’s very much focused on the physical treatment of that child. Sometimes issues do come up, particularly I've had one or two cases over the years where the family were really struggling financially and were asking the questions about, if a child is going to *********, how am I  going to get there? What accommodation do we have? Those kinds of things” [ID01 - clinician]  “I think unless they’re financially very, very well off they all struggle from what I’ve seen. The amount of times that we have to contact the hospital social worker or even the chaplaincy to ask for funding for these families while they’re in hospital. It costs such a lot of money to have a child in hospital, even just feeding yourself” [ID11]  “A lot of people don’t even want to ask for help do they? If you’re both working and your child’s in hospital, they might not necessarily want to say that they’ve only got £10 left in the bank until they get paid. But I know there’s a hell of a lot of people, especially in this day and age who are in that situation all of the time normally without having a child in hospital” [ID11]  “I was chatting to a family not that long ago at work, I was team leader and the mum was really  teary and I was saying to her, “Is there anything that we can do?” And she said, “I feel really embarrassed saying this but I’ve just I’ve got no money. I’ve got no money, I need…”  And I said, “Well, have you eaten today? Can we get you anything? Can I even just make you a cuppa and get you a couple of biscuits? What can I do to help?” And I ended up ringing the hospital social worker and saying, “This mum literally has no money and she’s not a resident…” It was a newborn baby but she’d been discharged from maternity services but she wasn’t from the local area” [ID11]  “We’re just so focused on PICU and fixing these kids physically, there’s something physically wrong with them, let’s get them fixed, let’s send them on their way, they get them off to the ward but we don’t actually think about the effects mentally, the financial strain, the physical illness has on the rest of the family. It’s just it’s bonkers sometimes that it just completely bypasses your mind, you have to actually think outside the box and think, “Well, what else is actually going on here with this family? What else can we do for them?” Rather than just saying, “Right, well, we’re going to give your kid the antibiotics, they’ll be fine.” There’s a million other things that come into play” [ID11]  “Some of our families are going to be beyond where we’re looking at poverty needs and lines. So when you’re talking about access and for surgical, if they’re the families that are coming in for surgery that’s been cancelled three times, how does that work? If they haven’t got family members to look after a sibling, then they’re having to pay out somewhere” [ID04]  “We’ve got one of the smallest populations of children within our geographical patch, but we’ve got the most deprived children, as well. So how does that offset each other, in terms of budgeting per capita? Because at the minute, it doesn’t. There isn't a scale of index, where you can say, “You are this deprived, so you will get this much money,” or, “Your region will get this much money, based on the amount of deprivation versus the amount of children that you’ve got, to provide the services.” [ID09]  “ID03: Even if you’re just talking about the cost of just surviving whilst on the wards, your parking, because they recently revoked the parking a couple of years ago and they’ve got this other scheme now where, if you apply in advance... This got me. “If you remember to apply in advance when your child is admitted acutely or even planned, because your head is everywhere else, oh, if you apply in advance, you can get a reduced rate on your weekly parking. Aren’t we good for doing that for you?” Where do families eat? How do they even afford to eat? In the [name of hospital], yes, you’ve got the different canteens and concessions,  but they’re all...  ID04: They’re all extortionate.  ID03: They’re all extortionate. I mean, I was sure that I should have taken out shares in [name of coffee shop chain]. I should have bought a [name of coffee shop chain] franchise in there because, honestly, I would have kept it going just myself.  “When it comes to night, there will be flexible charges with the taxis. There is never a constant charge for the taxis. If you want to come only one time, they will be charging £10. Sometimes they will be charging £20, £30, like that; it depends upon the period” [ID08]  “So this particular mummy had a few children, had a partner, was in a council house. Was managing okay, the mental health bucket was probably full but she was alright. That partner died of cancer, the mum was forced to take a different property for some reason. I can’t remember the exact reason why because it was some time ago, but was forced to take a house she couldn’t afford because that was what she so it was either that or be homeless. But she knew she was going to go into rent arrears but the social worker she said, “You must take that house because that’s what we’re offering.” I was like, “I don’t understand why you would force someone to take a property they can’t afford. I don’t get it […] Basically she was getting put further into poverty, yeah. And then so the social workers, I don’t know, there was just no logic, there was no care, there was no compassion, there was just, “You must take that property because that’s what I’m offering you.” And then it was like, I don’t know, that parent was seen as some sort of barrier for that person getting the job done and it’s just like they’re very just task orientated […] And then as it happens this mum had a baby that was really poorly and the baby did survive but she arrested. And she had all that other additional stress to deal with plus the fact that she knew she was probably going to end up being in rent arrears and have to move again with the children with no partner to support her” [ID12]  “I can’t access things that are to do with families. You have to go through a massive rigmarole to get any information about the social things” [ID12]  “She’s got children to think of and she’s just had a massive bereavement and she’s told, “No, you must go to this house because that’s what’s available.” “But I can’t afford the rent.” “Tough.” I don’t understand. She’s got no choice in life, it’s taking away her choice” [ID12]  “Again to me it goes back to I think health visitors are a lot better but it’s the social work. It’s as if they have all the answers and I think it would help if there was more communication between the two. So what I always find really, really frustrating is the fact that we don’t have any shared records between social work and healthcare” [ID12]  “And it always happens on a weekend or the middle of the night so you have to ring a duty team. Who then you ring them and then they don’t ring you back for however long it takes to get  the social worker. You can’t have a direct number to them for some reason, it’s like there’s some sort of… it’s like they’re magic and you can only get to them if you’ve got a password, it’s just…  But then usually I’m the nurse in charge or the team leader so helping the bedside nurse and it takes up so much of your time, hours and hours and hours. You have to document it all and I’m supposed to be in charge of a full unit of sick kids, it’s… it’s crazy” [ID12]  “So a patient might come from another district general hospital, they’ll come in and they’ll say, “There’s been domestic violence with that dad” and you’ll be like, “Right, well, is he allowed to  visit? What’s going on? Is he violent? What’s the problem?” and you have to do all those things that I said about ringing the duty team getting them to ring you back before you can find  anything out. And if it was just there on a shared thing so that you could see exactly what the problem was and if he was allowed to visit you could literally just look at it and not bother anyone” [ID12]  “It’s like a blockage, it’s so strange. Sometimes you’ll ring them up [social services], say their dad’s on the sex offender’s register or the uncle is or something, someone in the family, and you’re like, “Okay, so what have they done?” “Oh, I can’t tell you that, they’re just a sex…” and I’m like, “I need to know, there’s other children on this unit, there’s other families here. I don’t want to be putting the rest of this unit at risk. I’m not asking you because I’m being a gossip, I’m asking because I need to know.” But they just… sometimes they just don’t tell you it’s like […] I don’t know because we only interact when we have to so it’s not like you could go off and do a teambuilding thing or a meet and greet or some sort of networking day thing. I don’t know” [ID12]  “We usually do have a social worker and our hospital social workers are amazing. They’re much better and they can get access to things. And our safeguarding team can help but like I say, it happens in the middle of the night or on a weekend when none of those people are available so there needs to be somewhere where we can get some sort of access to that in my opinion” [ID12]  “I just think we have a lot, I feel that we have a lot more children who are from backgrounds that have got poor socioeconomic families. I can’t really put my finger on why it ends up happening because obviously I’ve not looked at all the research, they just come to us and we look after them and do whatever happens” [ID12] |
| **IMPACT ON CAREGIVERS ‘It’s trauma with fatigue’** | |
| Recurring themes –fear, stress, worry, exhaustion and fatigue, life on hold.  Draining.  Hospital experiences as traumatic.  Losing their trust of some members of hospital staff.  Highly stressful and emotionally-charged environments where everyone is aware that children are, or may, lose their lives.  Looking after caregiver's basic physical needs and mentions spiritual care (though no mention of sleep arrangements or mental health, trauma, socio-economic).  Uncertainty and not knowing what to expect making it worse ‐ this could be lessened if written information on process and signposting is provided.  Mentions inevitability that the health system is part of their lives, and this fact is inescapable.  Impact on caregiver, speaks of being in survival mode whilst having PTSD and having no other choice than to be ‘strong’.  Witnessing medical acts as hard ‐ came up in other interviews ‐ sense of powerlessness and lack of control in the worst possible moments.  Impact on siblings.  Frustration that caregivers were asked to leave the room their child is in so the medics can do a ward round.  Impossible worry of child having to face complicated and high-risk surgeries, multiple times.  Social determinants and mental health impact  Poverty, safe-guarding and social services  Importance of data sharing for safeguarding child patients ‐ important to liaise with social services to do this  Time wasted on chasing details for social services, especially on weekends ‐ overlaps with system barriers as refers to hospital-based social workers.  Nightmares and chronic shaking  Single parent dealing with child's illness ‐ highly stressful  Children at home with additional needs, caregivers exhausted from taking turns staying at the hospital. | “So me and mum are having to swap on a night, just so someone can stay with her. We have another daughter at home, who has additional needs, and it’s really difficult, that we’re having to do everything by ourselves. And obviously you work as a team. So if one of them is playing up at home, like my additional needs daughter, we’re both there to try and resolve that situation.  Like night feeds, because she’s only a month old, my other one. When you’ve gone from night feeds, doing it as a team, helping each other out when you're tired, to just being by yourself on a ward, with no sleep, staff coming in all the time, it’s extremely tiring and draining” [ID15]  “ID04: When my daughter went through her third cardiac surgery, I was eight-months pregnant, but we were expected to stay, and I was her main caregiver. Her dad is absolutely amazing, but I see things that he doesn’t see. So I slept on a bed, and it’s those things...  ID03: It’s not even a proper bed, is it? It’s a cot, a fold-out chair if you’re lucky.  ID04: No, no, a pull-down. Yes, I did have a chair a couple of nights. It’s understanding that, actually, and the big frustration around surgery is when planned surgery happens, all of those changes have been taking place and that child then goes into hospital. Then, they’ve been there all day, then the surgery is cancelled and you have to go through that again. So we have one family who has done that three times and it was very serious surgery that was required. Now, because that was the predominant issue, because they’d left it so long, the secondary issues were then becoming primary issues […] But you can’t do that to a family three times. We can’t do that. It’s not the parent, it’s the siblings. So you have a brother or a sister that’s terrified about what’s going to happen to their brother or sister, and then you’re saying it’s not happening. Then, you’re saying it's not happening again. So they’re going through that remit of emotions and then you’re into mental health support for that child”  “Yes, well, it’s trauma, with fatigue” [ID03]  ‘incredibly exhausting’ and ‘your life is on hold’ [ID03-04]  “It’s kind of expected, but I’ve got other children. I’ve got a job. Regardless, even if you didn’t have any of those things, no parents should be expected or have that worry that that’s what they’re going to have to do. Life is going to have be put on hold to then go in, and that is reflected across our families, isn’t it?” [ID03-04]  “So then families are dealing with primary issues, thinking, “Well, this was the primary issue, and now is it secondary?” Even when the surgery takes place, those other primary issues are now at a point where you’re never going to go back. We all understand emergencies happen and we all understand changes happen because emergencies and changes were our child once [laughs]” [ID04]  “That was the main concern, which is worrying us, so we will lift him up, and we just put him on our chest, and we just sit and sleep. For the past three months, no, it’s for the past six months we are doing it like this, and he has been sent for regular [school days 0:05:29]. And we even can't, if is here, we even can't go for work. And it’s a long waiting period to consult the ENT, that’s the main concern” [ID08]  “So, does that make any sense? I can’t tell it’s a sense because I already know that anaesthetists are a little bit busy there, and they have been struggling with a lot of strikes everywhere, so I don’t feel that it makes sense because it is a bit worrying from our end. It makes a lot of worry to us” [ID08]  “It’s mentally draining. Do you know what I mean? It’s just mentally draining. Because obviously, like I said, you end up doing it yourself, once one of them goes home. It’s like, “Right, okay.”” [ID16]  “And they were saying they don’t really like siblings on the ward, anyone under- I can’t remember the age, I don't know if it was 16 or what. But at the end of the day, it’s a children’s ward. Siblings, I believe, are just as important as parents. If not more important, because they don’t understand what is going on. One day their sibling is at home, okay, the next minute, they're suddenly in hospital and they don’t really get to see them” [ID16]  “We’ve had her [sibling] up twice, but on the first time, they said, “Look, you really shouldn’t be having her on the ward.” They just say because there are other poorly kids on the ward. But there are obviously other siblings there. I genuinely think it’s partly because of her additional needs, she can be quite full-on. And I think that could possibly be the reason. Like the other day, we had some family round, we went and sat in the family room. There were five of us, and fair enough,  they say only two, but that two includes one mum and one visitor, one dad and one visitor, or two visitors. You know what I mean?” [ID16]  “It’s definitely trauma, but they lose that sense of par- You get lots of conversations about people making decisions and people doing things and then telling you. So where do you stand as a parent? Actually, that decision lies with me unless... Make me understand a clinical decision because I’m more than happy...” [ID04]  “I actually was nearly admitted to a psychiatric unit because I had a migraine that was so severe from stress, on top of everything else, with nobody looking after me because, obviously, the nurses are there to do their job, to look after the child. They’re not there to look after the mother. They’re not there to look after the parent. They’re not there to make sure that she’s eating, she’s drinking, she’s sleeping, she’s not losing her mind. So then I had to be medicated, but then I was having to wrestle with: “Well, I need to provide ***’s care, but I’m going to be medicated, so what if I fall asleep, his machine goes off and someone doesn’t come in time?” All of this is catastrophising, but it’s a very, very normal trauma response to being in the situation with sick and poorly child”.  “The nurses on those wards know their craft inside and out, and like you say, 90% of them are phenomenal, but there would be certain nights where I would not sleep and I would be shaking in fear because I knew he was on shift and I knew that my *** wouldn’t get the care he needed”.  “Yes, and if you’re talking about access to services now, when you’ve lost that trust, it’s almost impossible to build that trust back up. If you’re preparing yourself, mentally, for what your child has to go through, you’re fighting a battle on both sides. You’re fighting a battle of this is your child and we know the worst-case scenario is that they’re not going to make it through surgery, and even the smaller surgeries. I read back on some of my messages and stuff, even to ***. It’s like, “God, it never gets easier, does it?” She’s had 13-hour surgeries and you think those are the worst, but actually, there’s no difference between a 13-hour surgery and a one-hour surgery” [ID04]  “I’m getting very personal, but I imagine this is very similar for a lot of families. Your trauma informs your future behaviour and you behave in a certain way. Now, I took on the role of the good patient, the good mother, and I got a lot of praise for that, inadvertently. “Oh, aren’t you doing well, Mum? Oh, you’re so calm. Oh, aren’t you doing...?” So even when I wanted to scream, even when I wanted to break down, I felt I couldn’t express that because I needed to be ‘best behaviour’, I needed to be good”. [ID03-04]  “Your mental health like a bucket. You can only have so much in before it starts to spill over and you can’t cope with everything in your life. So some of these families have got so much going on” [ID12]  “It’s the amount of people that have got sometimes it’s safeguarding issues as per se you’d call it a  safeguarding issue. It’s not necessarily that they’ve done anything to their child, it is just that they have not got- they might be being looked at with social services because of neglect. But in my opinion that’s because they haven’t got a lot of money, not because they want to neglect their child but because they haven’t got a lot of money. So they’re trying to think of other w- like they’re trying to make sure that everything else is okay so therefore their child’s a little bit neglected, you know what I mean?” [ID12]  “Working all hours or they’ve got loads of kids, a tiny house and not enough money and just stress all the time. In my opinion anybody in your life no matter where you get to is only a few steps away from that” [ID12]  “Even though she's been diagnosed two years, the seizures aren't something you're really made aware of. I didn't even know there was such a thing as diabetic seizures until it happened to us. So, generally speaking, we've always had okay experiences, but this one, in particular, recently, was pretty traumatic, if I'm honest. I don't know, I just even thought, "Just put her on the children's ward, just for a day. Just keep her here for a little bit, even 12 hours." Because obviously, coming home, like it was just traumatising. I couldn't even leave - take my eyes off her. That night, my mum was here and bathed the little one. The noise of the water going down the plughole, it made us think of the sound that she was making when I found her” [ID13_14]  “Because when it all happened, it's not really very funny now. But when I think about it, "Oh, my God." I was so traumatised. I just passed out and I started vomiting. So, they were having to see to me, while seeing to her. Because I was just in this- I can't even explain it, it was just so traumatising. I remember just feeling like my legs were going to go, and then I just went. And then, when I come around, and I was just vomiting, and vomiting, and vomiting” [ID13_14]  “I've been saying that I literally have- I've been having nightmares. Every little noise in the night. I get these unbelievable, uncontrollable shakes inside my body, like a tremor. It's completely changed everything. It has stopped her being able to go out with her friends because I was just so  scared it was going to happen again. I just think if somebody had sat us down, and went, "You know what? It might happen again, but you can't- but just know." There's been nothing, no  follow-up, nothing. So, here we are just self-learning how to just deal with it, and just hope for the best that it was a one-off” [ID13_14] |
| **IMPROVEMENTS** | |
|  | “I think the doctors should listen to the patients’ mental health more. Because worrying is not nice for anyone” [ID05 - YP]  “I feel like they should tell me when they’re going to do it, and make it as accurate as possible, and if they knew it would happen early, they should tell me a bit before so that I can actually prepare myself for the worry” [ID05 - YP]  “I'd probably just say move to a ward where there's more people who know about it. Even if it was the kid's ward, or a diabetic ward, I'd rather just move to somewhere where people know - people who learn about the stuff like that, can help instead” [ID14 - YP]  “I think I would have liked someone, like a diabetic specialist, or like a paediatrician, to come and say, "This has happened, and hopefully, it shouldn't happen again." Rather than it being just  unexplained, and not knowing the cause” [ID14 – YP, ED]  “Definitely shift change, handovers, parents should be allowed to sit in on that handover. Because then you're getting every piece of information, rather than what they want to disclose to you, if that makes sense” [ID16]  “More support for parents, to be fair. Clear communication. Meals included. If they're wanting you to be on the ward, they need to be supplying you with some sort of food, to be fair. Because you can’t give effective care if you're tired, if you're knackered, if you're starving. And you get more tired, you get lethargic, you get ratty. And when parents are unemployed and don’t have the money- Look at our region, it is one of the highest unemployment rates in the country” [ID16]  **“**I totally get that they are the experts, absolutely, and it’s like you just said, it should be more of a decision from what the child thinks, what the parent thinks, and what the doctor thinks, rather than just, “No, I'm the doctor, I know.” I'm not saying they don’t know, because they do know, but really it’s true that ******* … They went, “Ooh, it’s really swollen, it could have burst afterwards.” I didn’t say anything, I don’t want to argue, they worked hard those doctors” [ID06 - caregiver]  “I think that there needs to be regional oversight about referral pathways for dental care for children. We need to have equality in referral criteria. There needs to be a better mapping out of which services are open to which children. There needs to be a redistribution of specialist services out into other areas of the region, so not just centrally is what I mean. We need to have access to speciality service, even if that is just treatment planning, for children. We need to have more lists which have appropriate levels of staffing for children with comorbidities, so that they are getting the quality of care of their healthy counterparts. We need to, I think, have a recognition that dental pain and infection is just as important as pain and infection from any other part of the body, and perhaps can even be more debilitating because of its effect on eating, sleeping, concentration at school, ability to maintain weight, and also overall mood and wellbeing. We shouldn’t be the first people to be cancelled if there are shortages on the anaesthetic rota or  [recovery 00:17:43] rota” [ID15]  “We do have to think about how we utilise dental lists effectively because we know that dental is one of the most common reasons that children are admitted to hospital and, therefore, there is such a demand for the resource. Any list we get, we need to make sure that it’s utilised effectively. So we have the right number of patients on it, so we book the right number of patients on it, that has to do with planning from an experienced-surgeon point of view so that they waitlist patients for the appropriate amount of time. Also from a dental theatre coordinator point of view, so that if any patients cancel short notice there is a staff member available to refill them so that there is correct list management. That is not available in all services. Every service plans their list differently and they don’t all have access to a member of staff that can fill late-notice cancellations” [ID15]  “The other aspect of that is ensuring that every child that has a dental general anaesthetic has the most appropriate treatment so that it reduces their risk of having a repeat general anaesthetic. That has to do with the clinical assessment prior to the general anaesthetic. Ideally, they should all have pre-operative radiographs. Not all children in the region, that have a dental GA, will have dental radiographs. Sometimes it’s because they won’t cooperate but sometimes it’s because they’re seen as not a need that we’re going to go in and assess clinically at the time. However, in  theatre, there is not always access to radiographs or time to take radiographs built into the general anaesthetic episode so there are some children that have undiagnosed dental decay that is untreated during their general anaesthetic because they haven’t had the most appropriate assessment” [ID15]  “I think the recognition that there's… I know it’s more a paediatric intensive care setting but, really, for those low level HDU-level patients, I think there needs to be good provision for those kids to be moved to ********* and to be moved earlier so that they're in the right place to get the care they need at that time and, also, probably more importantly, so that they're in the right place if they deteriorate further” [ID01]  “There needs to be a lower threshold for ******* and, certainly, for ********** given the distances and time involved and delays that that brings in. I think that’s where our kids, at this end, are disadvantaged. They're just further away from those services and, therefore, to some extent, there's a reluctance for services to come out and get them sometimes” [ID01]  “One of the things that would help would be to have more staffing and to upgrade this to a Level 2 unit, which means we would have a chance to have one nurse to one or two children, to attend to those who are most critically unwell, which is often the case, to be honest. It is often about having a lot of low level acuity children on the ward but then having one who is really unwell, and probably doesn’t need the ICU, but certainly needs a lot of attention, which we are not able to provide” [ID02]  “They should really be giving you an option of like a four-hour slot or up to two hours or whatever, or you could have a slot or whatever” [ID06 – caregiver]  “How do we reset the balance, such that that specialist centre can be exactly that? And we make sure that all the provision that’s around it is set up in the correct way, to keep those specialist beds where they need to be, and utilised for what they need to be utilised for” [ID09]  “Hopefully that will change over time, as people understand the service better. But I think certainly the comms that went out about it, in my opinion, weren’t as good as they could have been. They weren’t as dynamic as they could have been, to tell people about, “What does this actually mean, in language that I understand?” Yes, I think it wasn’t handled as well as it could have been. And from a service, it could be a brilliant thing to showcase within the region, that we’ve got the only children’s urgent care centre. And it operates with nurses. And it’s all-singing, all-dancing, for this set of things that it does. And it’s great. And the staff that work there are wonderful, and the kids get a great deal when they go there. But I don't think it’s utilised yet as well as it could be, because of those barriers” [ID09]  “I would hope that I would never put somebody off coming, when they thought they have to. I would hope that I would educate them to come at the right time and empower them to understand when they're totally capable of looking after their little one at home themselves, for a certain length of time. And that’s again, about those safety-netting conversations and that kind of thing” [ID09]  “Whilst I very much feel that [tertiary centre] is the centre of excellence for all of the speciality services that they hold, they also have a general population to serve, as a local hospital. And if they're going to continue to do that, there needs to be provision in place that the children get what they need from that local general paediatric service. And I feel really strongly about that. That the provision and the skills aren’t given the time and money and facilities that they should be given. And that’s purely personal, that’s from what I’ve lived through, with the two service changes that we’ve been through. Equally, all of the things that we predicted would be problems, with the closure of [DGH 2], have been problems, and have been even bigger problems than we thought there would be. So I think there is a lot in listening to the people who are on the ground, with the people who are making the decisions, about service change. And I don’t think enough of that happens” [ID09]  “What we have to do is shift the mindset from, what do we need in critical care and surgery, to… Surgery is slightly different, because that will happen in a lot of different places, and are those pathways correct? Have we got the right people with the right skillset in the right places? Do we need to move those people, rather than moving the children? And I think there’s a lot in that, that we’re working on. But critical care needs to be almost ring fenced and protected. But the way to do that is to ring fence and protect the outer services that filter into it. So you keep the children where they need to be, until they need to be in a critical care bed” [ID09]  “There’s a longer-term plan of what we need to do and what we need to put in place, to make sure that the services that we’ve got are the right services in the right place” [ID09]  “I think that’s the social work one, and then there’s also the chaplaincy does provide some money for families for travel costs and food costs. But I’m not sure how you actually apply for that. The chaplains are really good though you could just ring them and say, “What is this fund that I’ve heard about?” but, yeah, it’s definitely there. But I think becau- I mean coming from my point of view not even knowing how to access that, if people don’t even know it’s there. So really just… maybe I suppose making it widely known amongst all of the staff, everyone, medical staff as well that if parents are in receipt of benefits or if parents… Not even that because I don’t want it to be that means tested type of thing because it’s expensive for everyone. But just that if they mention anything or for us to maybe ask them and say, “Will you have any financial worries while you’re in hospital? Is there anything we can help with?” [ID11]  “I guess just like a baseline indication of time. Because the surgeon did say, he said, "Oh, it's a very, very long waiting list." And I said, "That's fine." I said, "I'm not trying to push ****** up the list, just it would be really useful to have an idea, a ballpark." And he wasn't happy to offer that, and I can understand that, as well as elective stuff, he'll have more urgent stuff coming up, and repeat operations” [ID10]  “I know they're really busy, and it's already complicated enough, but could there have been a bit of an earlier warning shot, as you get closer on the waiting list that, "Okay, you're going to be the next two months." Or something just with a little bit of warning. Because I think it is a bit of a shock, and just having the preparation time would help” [ID10]  “I don’t know how it works at NHS England and all these big people that make all these big decisions but for such a huge geographical area we definitely could do with a bigger PICU. Certainly an HDU area within that PICU to look after the kids that just need that little bit of high dependency care that can’t be looked after on a ward” [ID11]  “I don’t really know what you can do about that apart from actually I’ve just thought, so in PICU we have advanced critical care practitioners who obviously are nurses by background but they are a constant person who are there all the time. So then you don’t have that, “Oh, no, they’ve got to learn their job.” They’re on the medical rota rather than the nursing rota and they obviously pass on their years of knowledge and expertise” [ID12]  “I feel when we’re talking about surgery, and we know that surgery is coming, it should be part of that conversation with that child so that parents aren’t put in that position where you’re actually attempting to drag your child out of the house because they’re having that emotional breakdown and you’re having to use your best actor face to not show that emotional breakdown because you’re feeling exactly the same. If you could just block all the doors, close all the curtains and  hide in the house ...” [ID04]  “Look for ways for them [CYP] to help to be as in control as they can be and to have autonomy over themselves because, like you say, you know it’s got to happen, but some very simple... Not simple in the sense of it, but it would be relatively straightforward because you’ve already got some of this information to create systems that then can just be used. Yes, it’s a big piece of work in the initial outlay” [ID04]  “I can’t access things that are to do with families. You have to go through a massive rigmarole to get any information about the social things […] If we did have more of a health and social care as one entity then you could access all that information and it would probably save man hours” [ID12]  “I do medical mediation as well, training for the staff. If that’s of any interest to you, so just to try and help with the stuff that you were talking about, to help us to have a better relationship with the families and if they’re traumatised to realise that that’s where the conflict comes from. And that it’s not actually like they’re not a difficult parent, it’s a difficult situation because they’re in an awful place” [ID12]  “I just feel like, for me, the main thing is, if a child attends A&E, and has to see past the initial assessment, there should be paediatric nurses or clinicians available. They shouldn't have to be on a ward with adult nurses who don't deal with paediatrics” [ID13] |
| **Participant suggestions on improving recruitment strategies for future studies**  Distribute information sheets to caregivers on wards at start of admission  Consultants meetings  Researcher/research nurse on wards to ask caregivers in person  Caregiver participants sharing study adverts with online parent support groups    Children’s hospices  Busyness as a barrier for healthcare staff  DEPICT research team  Identifying and contacting matrons for Oncology wards/day units  Research attends dental clinics and distributes study information directly  Contacting clinical directors for community dental services to distribute information sheets  Permission to access waiting lists to send study information to caregivers | “I would say start, and a reminder at the end, before you're discharged. Because then parents are aware that they’ve got this option at the start, and it gives them the thinking time, while they're on the ward, to gather some thoughts and information. And then, once they're discharged and settled or whatever, they can pass on all that relevant information to you” [ID16, caregiver]  “The other thing just to mention for your study, we did have a consultants’ meeting yesterday where I told everybody I was having this phone call today and I think everybody else keen to speak to you at some point as well” [ID01]  “I thought it would be good to ask people who I know that they had experience with critical care rather than just having posters on the walls” [ID02]  “I can’t think of anyone off the top of my head, personally, but what I’ll do is I’ll see what I can do and then I’ll email your details to anybody that I come across. I’ll put it onto my own group. That’s fine” [ID05_06]  “I know you’ve got your poster, I saw the poster in, what do you call it, in the adult room, but I don't know whether you can, or you're allowed to, but have you not thought about putting it on a ***** **** group, you could say, “Has your child?” like on Facebook or wherever, like advertise.  In surgery, obviously with ICU it’s obviously more [ ___ 0:32:24] as well, “We’re looking to make improvements,” like you did really, or put it on social media, over Facebook. I run a group on Facebook for ********** mums and dads, ********** ********, a home ed group as well up ****** ****, groups like that. I don't know anyone personally who’s up here for it” [ID05_06]  “******* ******* is a hospice that will deal with only children. Whether a child or parents need respite and the child needs to go there, or whether the child has ongoing end-of-life care,  etc., that is what they deal with and that is what they handle. Now, they are absolutely lovely people. I met one of the ladies who runs it, again not that long ago. But they would probably  give you more insight to people not being able to attend appointments or not being able to get to A&E, or the medical expensive equipment at home and stuff like that. They would probably be a good group of people to chat to” [ID07]  “I think it's really tricky, and I don't- I mean, I could say that I could use word of mouth, but I don't work in hospitals. So, I'm no use. But I think it's really difficult. I don't know the best way, but I can imagine it's really tricky, and then everyone's so busy. Everyone's so busy, but obviously, you want the healthcare professionals' views. So, I don't even know” [ID10]  “I’ve thought of another one as well actually, you know at [hospital transport service] we ran a study called the DEPICT Study. It was parents and carers experiences of being picked up at the local hospital, the [hospital transport service] journey and then arriving at paediatric extensive care […] So get in touch with them because if the people who were really forthcoming within that study… because I did a lot of the bits for that study, I was the lead nurse if you like for a while. And some of the caregivers were actually really desperate to tell their story, they wanted to talk about it and they wanted… so if there’s people like that. I mean I know they can’t give details out but if they could contact and say would you be interested in completing this?” [ID11]  “So Ward 14, which is their oncology day unit and Ward… now where do they go from Ward 3, because Ward 3 have got a day unit attached to them as well for the BMT patients” [ID11]  “The clinical directors of the community dental services, they would be able to do the same thing for you if you gave them your participant information leaflets, they could do that […] What I could do is email you who I got your details from, because that group has all the clinical directors of the community dental service so she would be able to get you the right contact details” [ID15]  “I think the best place for recruiting is usually our clinics, to be honest. Even if you had leaflets that you wanted to give us, then we could hand them out when we identify a patient that’s  getting put on a general anaesthetic list” [ID15] |
| **Recommendations** | |
| Health system | Lower thresholds for transfer to city hospitals from rural areas.  Smoother transitions between transfer processes.  Recognition and realisation of the situation in rural areas.  Upgrading to Level 2 units in rural areas.  More funded posts and physical space, capacity and resources to meet demand.  Advanced critical care practitioners as mentors to junior medical staff on PICU to pass on knowledge and expertise.  Hospital staff to be trained in trauma-informed approaches if not already.  Providing an indication (e.g. a week or 48 hours) or advance warning of when surgery is likely to occur so caregivers can plan.  Improved hospital accommodation for caregivers.  Providing hospital food for caregivers staying at the hospital with their child.  Reducing parking costs for outpatients and inpatients.  Hospital staff signposting caregivers and siblings to mental health support.  Providing trauma-informed counsellors on the unit for caregivers and staff to access.  Including play therapists and play specialists as emotional support conduits.  Protecting statutory equipment provision.  Regional oversight of dental paediatric pathways.  Mapping of dental services across region for NHS referrers to locate nearest services for patients.  Redistribution of dental services across regions, not just centrally.  More access to paediatric dental specialists.  More dental lists to meet demand and reduce waiting times.  Recognition that dental pain is just as important as pain and infection from any other part of the body.  Skilling up and empowering caregivers to build confidence, especially when leaving paediatric critical care to return home without the necessary equipment.  Providing access to trained and qualified PAs.  Addressing the cost of attending/staying in hospital and offering financial and sustenance assistance, especially for those living on a low-income.  Ensuring specialist beds protected.  Strategic approach to providing education and training across the region.  Medical mediation training for staff.  Role of hospital social worker in addressing barriers to access.  Joined up data systems between hospitals and social care to understand social determinants to optimise care.  Dental list management as important.  Dental waiting lists planned by an experienced surgeon point of view so patients waiting for an appropriate amount of time.  Every CYP should have dental general anaesthetic to reduce risk of repeat attendance (ascertained in clinical assessment prior to general anaesthetic).  All CYP should have pre‐operative radiographs (to identify undiagnosed dental decay ‐ so improved  assessments). |
| Communication | Empowering CYP patients “*look for ways for them to help to be as in control as they can be and to have autonomy over themselves*”.  Providing accessible and creative infographics for children and young people so they can understand condition/treatment/situation better.  Familiar hospital staff who explain procedures to children and caregivers in an accessible manner rather than caregivers with no medical training.  Listening and supporting the needs of caregivers and putting on events like coffee mornings.  Campaign to improve public understanding of regional system/service to demystify clinical pathways.  Providing access to legal services so caregivers know their welfare rights and entitlements.  Caregivers knowing in advance what the protocols are in paediatric critical care and surgery for children.  Providing caregivers with a clear understanding of clinical roles involved and who is responsible for what.  Helping caregivers to understand clinical reasoning and decision-making.  Allowing caregivers to sit-in during staff handovers.  Connecting caregivers with a wider network of families living with similar health conditions.  Listening to staff on the ground, consult with them before making restructuring decisions. |
